# Mechanosensitive Non-Coding RNAs in Cardiovascular Disease: Mechanisms, Therapeutic Targeting and Cardiac Delivery

**DOI:** 10.3390/cells15211952

**Published:** 2026-10-27

**Authors:** Irene Fancello, Veronica Candino, Daniel Gamo Gonzalez, Maurizio Pesce, Gloria Garoffolo

**Affiliations:** 1 Unità di Ingegneria Tissutale Cardiovascolare, Centro Cardiologico Monzino, IRCCS, 20138 Milan, Italy; irene.fancello@cardiologicomonzino.it (I.F.); veronica.candino@cardiologicomonzino.it (V.C.); daniel.gamo@cardiologicomonzino.it (D.G.G.); maurizio.pesce@cardiologicomonzino.it (M.P.); 2 Department of Medicine, Libera Università Mediterranea (LUM) University, 70010 Casamassima, Italy

**Keywords:** non coding RNAs, mechanotransduction, cardiovascular diseases, cardiac delivery

## Abstract

Non-coding RNAs (ncRNAs) have emerged as major regulators of gene expression and cellular homeostasis, with growing evidence supporting their involvement in cardiovascular physiology and disease. This review summarizes the classification, mechanisms of action, and functional roles of ncRNAs, with particular focus on mechano-ncRNAs involved in cardiovascular remodeling and fibrosis. Different classes of ncRNAs are discussed in relation to their contributions to vascular, valvular, and myocardial homeostasis, as well as to pathological processes including inflammation, endothelial dysfunction, and fibrotic responses. Special emphasis is placed on biomechanical stimuli, including increased matrix stiffness, cyclic strain, and altered shear stress, which can modulate ncRNA expression and activity through mechanotransduction pathways. Notable examples of mechano-ncRNAs include *miR-21*, *miR-29*, *miR-132*, *MIAT*, *MALAT1*, and *MEG3*, which have been implicated in maladaptive cardiac remodeling and fibrosis. Concurrently, we highlight the importance of technical advances in ncRNA discovery and cardiac-specific delivery systems, including viral and nanoparticle-based vectors, for the development of ncRNA-based therapeutic strategies with potential for clinical translation. Overall, ncRNAs are shown to represent promising biomarkers and therapeutic targets in cardiovascular diseases, particularly in mechanically driven pathological conditions. However, challenges related to delivery specificity, stability, off-target effects, and safety remain and will need to be addressed to enable the clinical translation of ncRNA-based therapies in precision cardiovascular medicine.

## 1. Introduction

In the cardiovascular system, ncRNAs regulate important cellular processes, including metabolism, proliferation, differentiation, apoptosis, and responses to the extracellular environment [1,2]. Consequently, several ncRNA species have been shown to influence the pathophysiology of the heart and vessels, acting either as disease drivers or protective factors, as well as diagnostic and prognostic biomarkers [3].

Biomechanical extracellular stimuli are sensed by cells and converted through mechanotransduction pathways into phenotypic changes that may ultimately lead to cardiovascular dysfunction. In this context, numerous ncRNAs are regulated by mechanical stimulation and have been implicated in the onset and progression of cardiovascular diseases (CVDs), making them attractive therapeutic targets.

This review summarizes current knowledge on the interplay between cardiovascular ncRNAs and mechanosensing, with a particular focus on mechano-ncRNAs involved in promoting or limiting adverse cardiovascular events. Rather than providing a broad catalog of ncRNAs associated with cardiovascular disease, the distinctive perspective of this review is to integrate three interconnected dimensions that are often considered separately: the biomechanical regulation of ncRNAs, their functional integration within canonical mechanotransduction pathways, and the strategies required for their therapeutic modulation in cardiovascular tissues. It therefore discusses how specific mechanical cues, including matrix stiffness, cyclic strain, pressure overload, and altered shear stress, can shape ncRNA expression and activity, and how these molecules may in turn act as effectors, modulators, or molecular memory elements of mechanotransduction. It also examines experimental and computational approaches for identifying novel mechanosensitive ncRNA signatures to support target and biomarker discovery. Finally, the review critically evaluates the current strategies for targeted cardiovascular delivery aimed at enabling clinically applicable therapies based on the modulation of ncRNAs. By connecting mechanobiology, ncRNA regulation, target discovery, and cardiac delivery within a single conceptual framework, this review aims to define mechano-ncRNAs as a distinct translational interface between cardiovascular mechanobiology and precision RNA therapeutics.

## 2. Non-Coding RNAs: Biological Relevance in Cardiovascular Physiology and Pathology

At least 80% of genomic DNA in higher eukaryotes is actively transcribed, yet less than 3% of the resulting transcripts encode for proteins, indicating that the vast majority of transcribed genes give rise to non-coding RNAs (ncRNAs) [4]. By definition, ncRNAs do not result in a protein product, although some transcripts may be associated with the production of micro-peptides [5,6]. However, their most distinctive feature is that their biological function is largely independent of protein translation [7].

**Figure 1:**
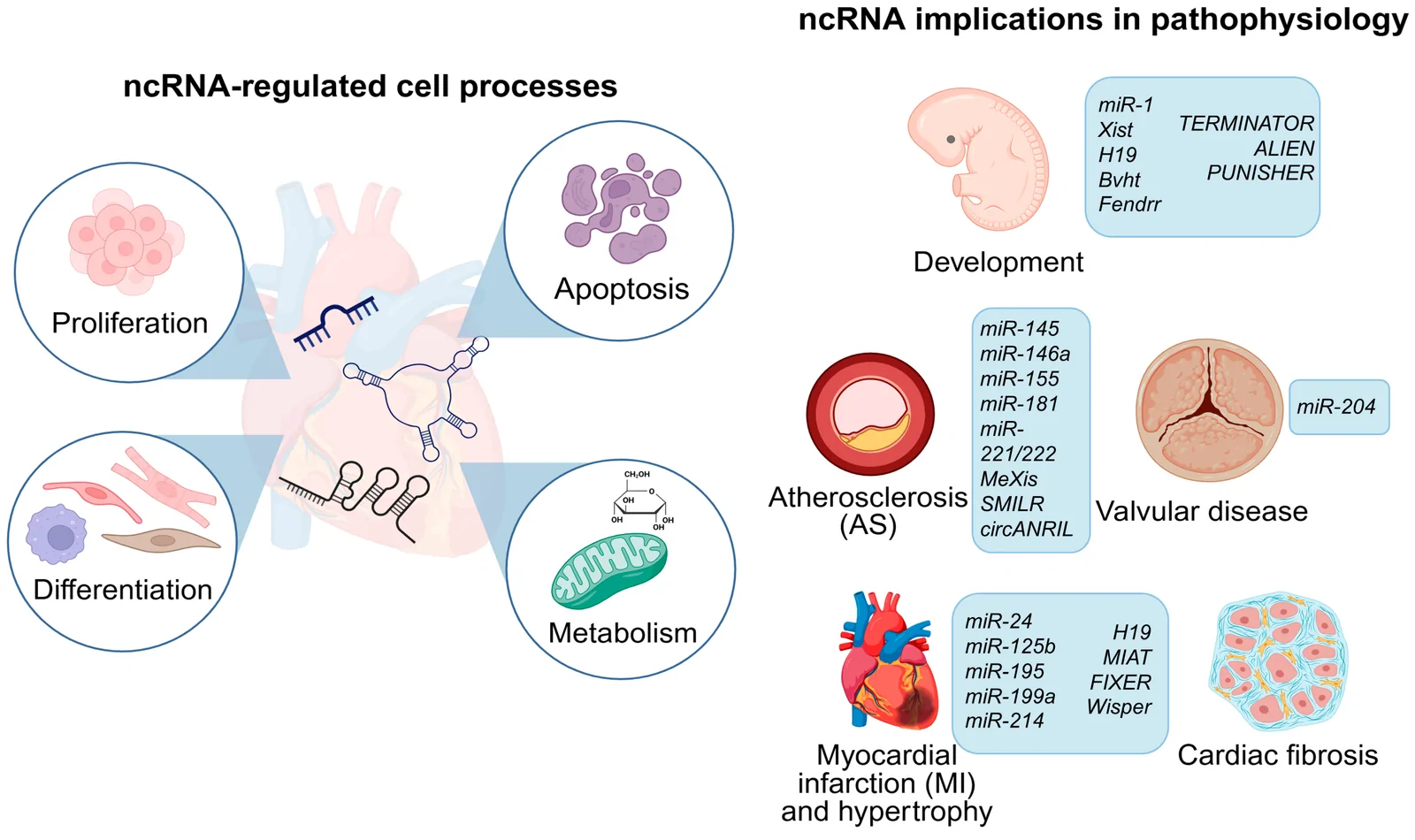
Impact of non-coding RNAs in cardiovascular development and pathology. In cardiovascular cells, ncRNAs affect cellular processes such as proliferation, apoptosis, differentiation, and metabolism. In turn, this has implications for the pathophysiology of development, atherosclerosis, valvular disease, myocardial infarction, hypertrophy, and cardiac fibrosis. Examples of ncRNAs involved in these processes are listed in the blue boxes.

Non-coding RNAs are known to affect complex molecular networks involved in cell proliferation, differentiation, apoptosis, and metabolism (Figure 1), thus playing a central role in the onset of several human pathologies, including cardiovascular diseases (CVDs). Altered expression of ncRNAs was found to be associated with different CVDs such as atherosclerosis, valvular disease, and heart failure due to ischemic damage, hypertrophy, or fibrosis (Figure 1).

Non-coding RNAs include several subtypes, classified by length, structure, and function. ncRNAs shorter than 200 nt can be classified into microRNAs (miRNAs), small nuclear and nucleolar ncRNAs (sncRNAs), PIWI-interacting RNAs (piRNAs), Y-RNAs, and tRNAs, while ncRNAs longer than 200 nt are called long non-coding RNAs (lncRNAs) [8,9]. Another class of ncRNAs is circular RNAs (circRNAs), of size >100 nt [10].

The biology of sncRNAs, piRNAs, Y-RNAs, and tRNAs has been reviewed previously [11,12,13,14] in cardiovascular research. Owing to the large body of literature available, this review does not aim to provide a comprehensive overview, but rather summarizes the most relevant and impactful studies involving miRNAs, lncRNAs, and circRNAs, and their role in both cardiovascular pathophysiology (Figure 1) and, in a later section, response to mechanical stress.

### 2.1. MicroRNAs

**Figure 2:**
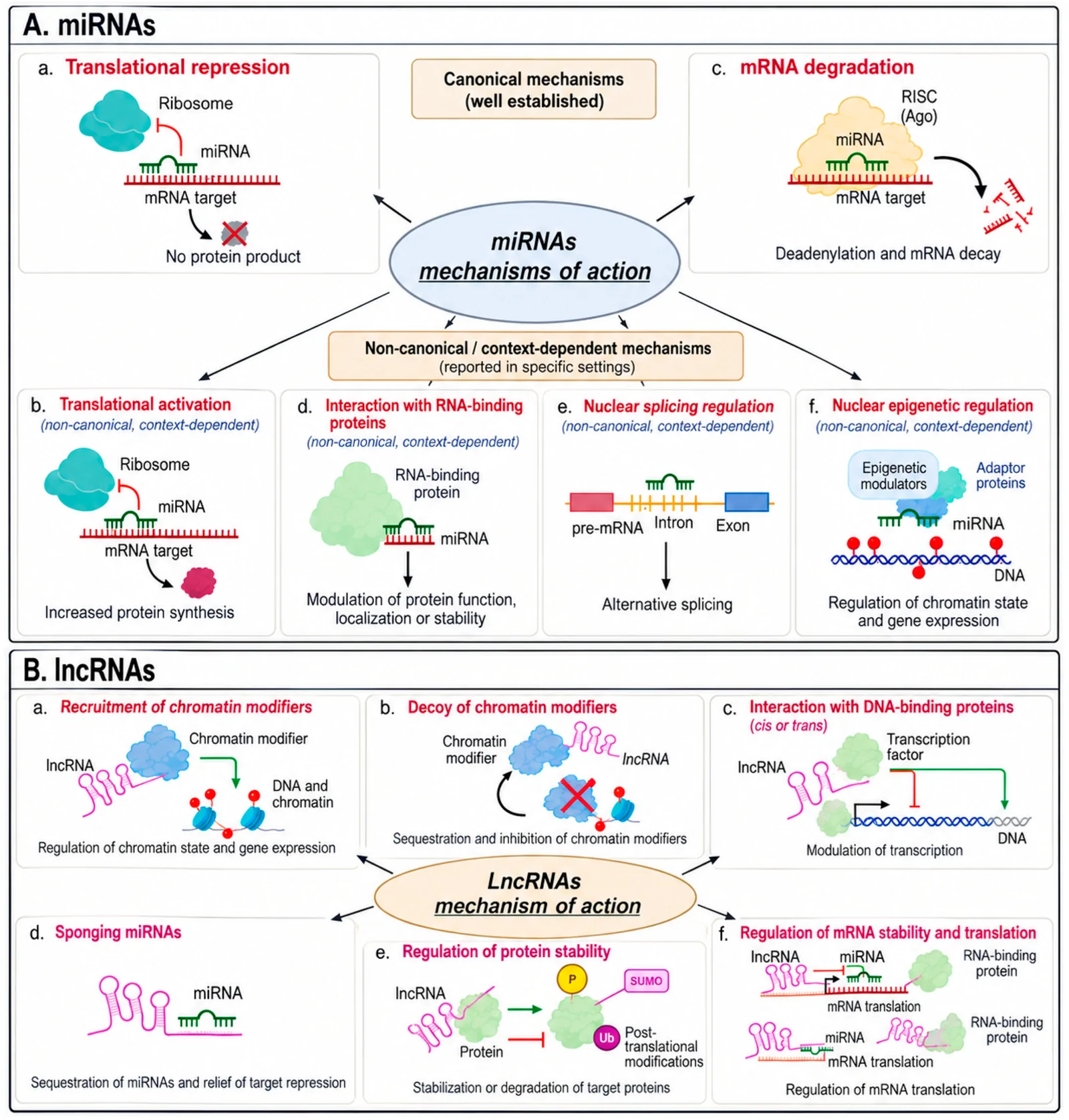
Mechanisms of action of miRNAs (**A**) and lncRNAs (**B**). miRNAs exert several functions through canonical or non-canonical mechanisms: partial binding of miRNA to its mRNA target can hamper ribosome binding and repress translation (**a**), or, conversely, recruit factors and ribosomes to activate translation (**b**); (**c**) complete binding of miRNA to the mRNA induces target cleavage and degradation; (**d**) miRNA binding to regulatory RNA-binding proteins can act as decoys and control protein function; in the nucleus, miRNAs can interfere with mRNA splicing processes (**e**), or recruit protein modulators to affect chromatin accessibility and regulate the epigenome (**f**). (**B**) In the nucleus, lncRNAs can have different roles: (**a**) lncRNAs can recruit chromatin modifiers to the gene promoter in order to activate or suppress transcription; (**b**) lncRNAs can act as decoys for chromatin modifiers to sequester them from the gene promoter; (**c**) lncRNAs can directly interact with DNA-binding proteins, including transcription factors, to regulate transcription. In the cytoplasm, lncRNAs can affect miRNA or protein stability, for instance by acting as miRNA sponges (**d**) or by inducing protein modifications (e.g., ubiquitination, phosphorylation) (**e**). LncRNAs can directly modulate mRNA stability (**f**) through two mechanisms: direct interaction with mRNAs to protect target mRNAs from miRNAs or RNA-binding protein (RBP)-driven degradation, or by sequestering miRNAs or RBPs to avoid their interaction with the target. Symbols: green arrows indicate activation/promotion; red blunt-ended lines indicate inhibition; black arrows indicate the resulting outcome; red dots on DNA represent epigenetic marks; miRNAs are shown in green, lncRNAs in magenta and mRNAs in red.

MicroRNAs are short transcripts of 19–23 nt in length that generally interact with complementary sequences on the 3′ UTR of target mRNAs, affecting their stability and translational output. Mature miRNAs are produced through a finely regulated process occurring in the nucleus and later in the cytoplasm [15]. Mammalian miRNAs are initially transcribed by RNA polymerase II/III, producing pri-miRNAs, which are cleaved in the nucleus by Drosha/DGCR8, releasing stem-loop precursors named pre-miRNAs. Upon cytoplasm exportation, further processing by Dicer and the Argonaute/RISC complex allows mature miRNAs to be obtained. The final miRNA then exerts its canonical function by directing the RISC complex to recognize and negatively regulate mRNA targets. Additional non-canonical miRNA functions instead involve positive translational control of mRNAs, as well as protein binding, splicing, and epigenetic regulation (Figure 2A). Imperfect complementarity is typically linked to mRNA translational modulation, resulting in the repressed or, less often, activated production of the corresponding protein (Figure 2(Aa,b)) [16]. Perfect matching triggers a stronger effect, inducing mRNA cleavage and thus degradation (Figure 2(Ac)) [17]. In addition, miRNA can further affect transcription and translation by acting as decoys for regulatory RNA-binding proteins (Figure 2(Ad)) [18]. The roles of miRNAs may extend beyond mRNA control, since they were detected in different subcellular and extracellular compartments. Outside of the cytoplasm, nuclear miRNAs can act as splicing or epigenetic regulators (Figure 2(Ae–f)) [19]. Several extracellular miRNAs have been detected in biological fluids, where they circulate within extracellular vesicles (EVs) and other cell-derived lipidic vesicles of various sizes that mediate paracrine and systemic intercellular signaling [20].

MiRNAs are important regulators of cellular phenotypes, impacting proliferation, differentiation, apoptosis, and metabolism, for example [15]. They are particularly relevant in the cardiovascular context because of their implications in development, regeneration, and pathophysiology of the heart and vessels. In fact, several miRNAs constitute the small ncRNA signature of these tissues in humans, where their alterations may regulate CVD development and progression [3,21]. Moreover, multiple miRNAs were found in the circulating blood and correlated with CVD diagnosis or prognosis, making them potential new disease biomarkers [22].

The role of miRNAs in cardiac development is exemplified by *miR-1*, which is pivotal for cardiac morphogenesis, affecting electrical conduction and cell cycle control in cardiomyocytes [23]. Several miRNAs modulate the function of endothelial cells (ECs), vascular smooth muscle cells (vSMCs), and macrophages, and have been found to be particularly implicated in lipid accumulation and inflammation in atherosclerosis. In ECs, for instance, a plethora of miRNAs, such as the *miR-181* family, *miR-155*, and *miR-221/222*, were found to suppress inflammatory responses through different intracellular pathways [24,25]. In macrophages, *miR-146a* modulates inflammation and plaque deposition by inhibiting TLR4-dependent intracellular signaling, resulting in a reduction of intracellular LDL cholesterol and secreted cytokines [26]. A notable example in VSMCs is represented by *miR-145.* While this miRNA is known for its cardioprotective function in cardiomyocytes [27], in VSMCs it is crucial for differentiation and phenotype switching, and it was found to be downregulated in atherosclerosis and arterial injury models [28].

Valvular heart diseases are characterized by extensive remodeling processes involving both protein-coding and non-coding genes [29]. In particular, several studies have indicated miRNAs as central regulators of aortic valve calcification and mitral valve stenosis [30,31,32,33]. For instance, in valve interstitial cells (VICs), *miR-204* represses Runt-related transcription factor 2 (RUNX2) and inhibits the expression of osteoblast-related genes, thus reducing the pro-calcific differentiation [33]. Consistent with its role, *miR-204* is negatively correlated with human stenotic valve disease [34].

The pathophysiological processes driving heart failure and cardiac hypertrophy are also subjected to complex miRNA modulation, and increasing evidence supports an effector or diagnostic/prognostic role of microRNA in adverse cardiac remodeling [35]. For instance, hypoxia-related *miR-210* is known to promote cardiac fibrosis induced by pressure overload [36]. *miR-24*, *miR-125b*, *miR-195*, *miR-199a*, and *miR-214* were found to be upregulated in patients with end-stage heart failure, thus working as important biomarkers to track the progression of the disease [37]. Information on their possible role as effectors of the disease has yet to be gathered, given the lethal phenotype resulting from their gain-of-function in vivo, but it was possible to observe that *miR-195* overexpression drives disorganization of cardiomyocytes and heart failure in transgenic mice [38]. These examples suggest that although many processes can be going on at the same time during disease progression, the induced alteration of a single microRNA is often sufficient to develop a pathological phenotype.

### 2.2. Long Non-Coding RNAs

More than 30,000 RNAs in the human transcriptome [39] account for non-coding transcripts of length >200 nt, classified as long non-coding RNAs.

Similar to mRNAs, mature lncRNAs have 5′ 7-methyl guanosine (m7G) caps, 3′ polyadenylated tails and undergo alternative splicing, creating several possible isoforms. Unlike them, they generally exhibit low abundance and evolutionary conservation [39]. As all non-coding transcripts, they are typically considered not to lead to the production of a protein product, but some possess small Open Reading Frames (sORFs) encoding short functional microproteins [40]. So far, lncRNA-derived microproteins have only been described as implicated in cancer regulation [5,41], but their putative roles in human diseases may be wider.

LncRNAs are generally known to exert their regulatory activity by working on different cellular levels depending on their sequence and localization: on the chromatin or DNA, on other RNAs, or on proteins (Figure 2B). These regulatory transcripts include *cis*- and *trans*-acting elements that control transcriptional or translational processes, regulating, for instance, mRNA localization, stabilization, or translation [3,42].

After transcription by RNA polymerase II/III, lncRNAs initially undergo splicing. In most cases, this leads to tethering to the chromatin or nuclear domains, which keeps them sequestered in the nucleus [43,44]. Here, nuclear lncRNAs can intervene in nuclear organization or gene expression control, for instance by affecting chromatin accessibility (Figure 2(Ba,b)), activation of enhancers or recruitment of the transcriptional machinery on local or distal genes, by binding directly to the DNA or indirectly through other proteins (Figure 2(Bc)) [45]. Alternatively, other lncRNAs can be exported to the cytoplasm and possibly assigned to different subcellular compartments or associated with RNA-binding proteins. Cytoplasmic lncRNAs are typically involved in mRNA turnover and accessibility to the ribosomes, as well as in protein stability and recruitment (Figure 2(Bf)) [46,47]. Importantly, cytosolic lncRNAs can endogenously compete with other regulatory RNAs (Figure 2(Bd)). For example, miRNAs can be sequestered by complementary binding (e.g., lncRNAs acting as miRNA “*sponges*”), reducing their availability and, in turn, indirectly regulating the levels of the target mRNAs [48]. Another relevant role of lncRNAs, either in the cytoplasm or in the nucleus, is the scaffolding of RNA-interacting proteins (Figure 2(Be)). The interaction between specific lncRNAs and big protein complexes through one, or possibly more, proteins can activate or modulate important cell functions, including signal peptide recognition or histone reorganization [49].

The classification of lncRNAs is not standardized, and it often depends on experimental features: for instance, it defines antisense (AS-lncRNAs), intergenic (lincRNAs), intronic, enhancer RNAs (eRNAs) based on genomic location, and transcriptional guides, protein scaffolds, decoys, and competing endogenous RNAs (ceRNAs) based on their molecular function [50]. The complete mechanistic classification has already been reviewed elsewhere [51] and falls outside the scope of this review.

Several lncRNAs have broad implications in mammalian development and cancer, such as *Xist* or *H19* [52,53]. In particular, a variety of lncRNAs have been found to significantly influence cardiac development. Notable examples are Braveheart (*Bvht*), *Fendrr, TERMINATOR*, *ALIEN*, and *PUNISHER*, which regulate cardiovascular lineage commitment and development of the heart and body wall [46,54,55,56].

Remarkably, specific lncRNAs can be involved in more than one regulatory mechanism depending on the context. For example, the lncRNA *H19* is also known to regulate the pathophysiological processes driving cardiac hypertrophy, such as ECM deposition, apoptosis, inflammation, and calcium flux [57,58].

LncRNAs, like miRNAs, can be correlated with, or work as, effectors or biomarkers of atherosclerosis. For instance, lncRNA *MeXis* controls cholesterol overload in macrophages by enhancing the expression of the cholesterol efflux regulator *ABCA1*. In turn, its suppression induces cholesterol retention in vivo, ultimately promoting atherosclerosis in mice [59]. Another example in VSMCs is *SMILR*, whose expression was found to be upregulated in proliferating and migrating smooth muscle cells, as well as in serum samples of patients with unstable atherosclerotic plaque [60].

Pathological ischemic and non-ischemic events may drive cardiac fibrosis, a process where activated fibroblasts act in concert with resident immune cells to establish a cycle of chronic inflammation and dysregulated ECM deposition. A multitude of lncRNAs are involved in the regulation of this process. For example, in mice, the lncRNAs *MIAT*, *FIXER*, and *Wisper* were found to be associated with the development of cardiac fibrosis [61,62,63], and their modulatory role was proven by direct experimental approaches. Indeed, depleting their levels individually in mouse models of MI was effective in attenuating MI-induced fibrosis and improving cardiac function [61,62,63].

### 2.3. Circular RNAs

A newly identified class of ncRNAs is represented by circular RNAs (circRNAs), which are structured as covalently closed continuous loops [64]. This circular structure, generated through back-splicing events, protects them from ribonuclease degradation, conferring high stability [65]. Although initially considered by-products of aberrant transcription or genomic noise, circRNAs are now recognized as important regulators of gene expression [64]. Their high abundance, stability, and evolutionary conservation across species suggest key roles in the modulation of cell fate and function. Mechanistically, circRNAs regulate gene expression by interacting with RNA-binding proteins to form ribonucleoprotein complexes, by influencing mRNA splicing, or by acting as molecular sponges for miRNAs [66,67].

CircRNAs are still relatively poorly studied, but recent evidence is showing their potential as therapeutic targets for cardiovascular diseases including atherosclerosis, MI, and heart failure [67]. Moreover, their detection in a variety of human biofluids has highlighted their utility as biomarkers in several contexts [68].

For example, in the context of atherosclerosis, *circANRIL* has been identified as negatively correlated with atherosclerotic severity, and a protective role against disease progression has been suggested through modulation of rRNA maturation [69]. However, its role was later revealed to be highly context-dependent, according to the specific cell types involved and disease phase, as underlined by recent transcriptomic studies [67]. In fact, *circANRIL* has been labeled as a disease driver during advanced stages in synthetic vSMCs and macrophages, where inflammatory stimuli are prominent, and a disease protector during early stages in ECs and contractile vSMCs [67].

Collectively, these studies illustrate the wide variety of functions of ncRNAs in cardiovascular diseases (CVDs), which can be highly dependent on cellular context and disease stage. Since the cardiovascular system is continuously exposed to dynamic biomechanical forces that profoundly influence the behavior of vascular and cardiac cells, understanding how these stimuli regulate ncRNA networks will be instrumental for the development of more selective diagnostic and therapeutic approaches based on mechanoresponsive ncRNAs.

## 3. Mechanosensitive Non-Coding RNAs in Cardiovascular Diseases

### 3.1. Mechanotransduction Pathways

The role of mechanical forces in cardiovascular pathologies is not limited to cellular differentiation or isolated gene expression changes, but rather underpins fundamental mechanisms driving disease progression across multiple conditions. In heart failure, for instance, pathological changes in ECM stiffness and myocardial wall tension contribute to maladaptive hypertrophy and fibrosis [70]. Acute myocardial infarction involves abrupt alterations in mechanical load and perfusion, which influence both immediate tissue damage and long-term remodeling. In atherosclerosis, disturbed or oscillatory shear stress at arterial bifurcations promotes endothelial dysfunction and localized plaque development [71]. In arterial hypertension, increased vascular wall stress and sustained pressure overload activate mechanotransduction pathways in ECs and vSMCs, leading to vascular remodeling and increased stiffness [72]. Across these disease contexts, non-coding RNAs serve as key intermediaries that integrate mechanical cues into gene regulatory responses, influencing inflammation, fibrosis, vascular tone, and cellular phenotype.

Therefore, the development of new therapeutic targets and diagnostic biomarkers for mechanically induced CVDs can be achieved by deepening our understanding of the role of ncRNAs in physiopathological processes involving mechanotransduction.

**Figure 3:**
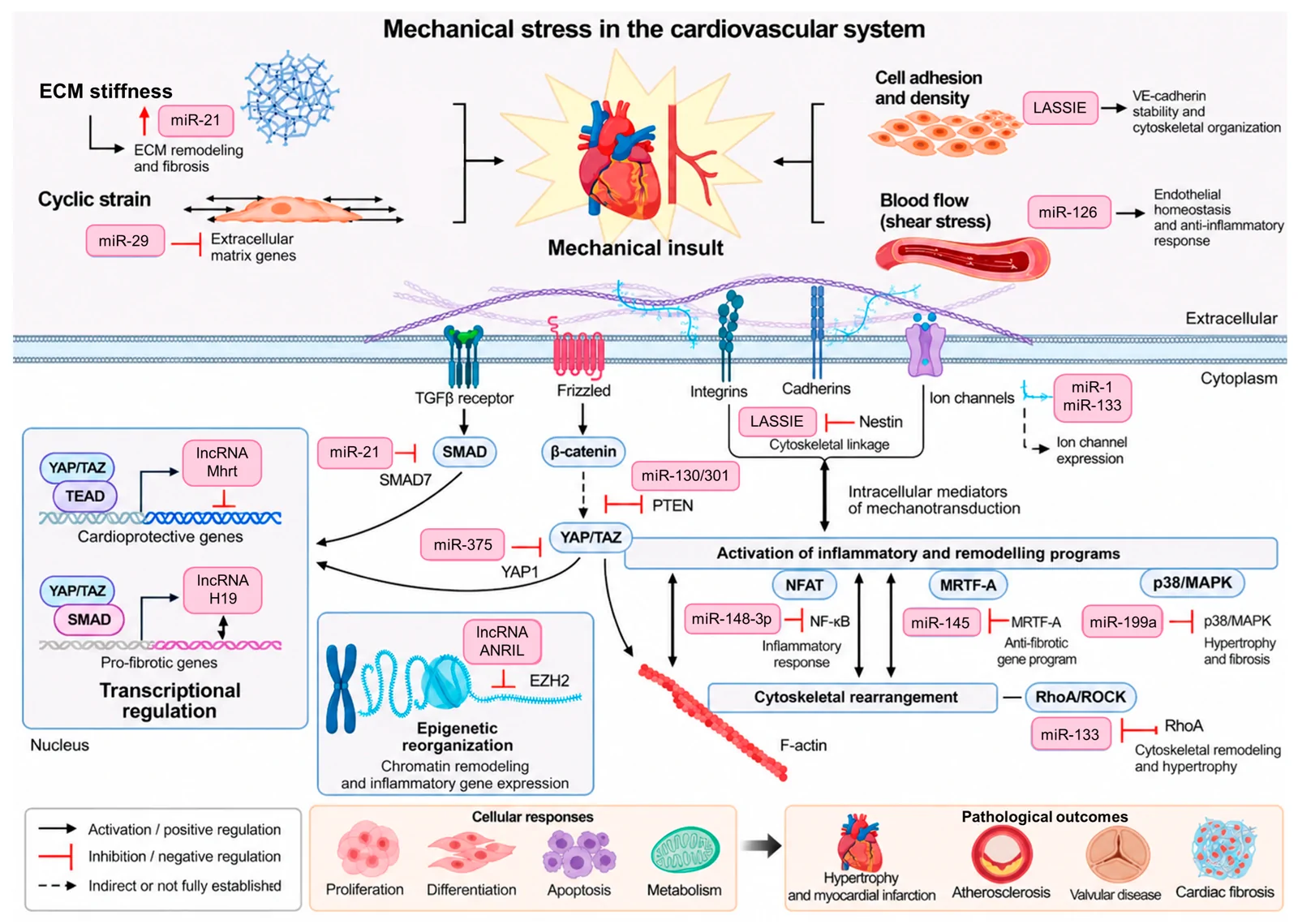
Mechanobiology and ncRNA regulation in cardiovascular diseases. Mechanical stimuli in the cardiovascular system, including ECM stiffness, cyclic strain, cell adhesion and density, and blood flow, are sensed through membrane and cytoskeletal mechanosensors and translated into intracellular signals involving pathways such as TGFβ/SMAD, β-catenin/YAP/TAZ, NFAT, MRTF-A, p38/MAPK, and RhoA/ROCK. Representative mechano-ncRNAs are integrated at their experimentally characterized molecular nodes, highlighting their ability to modulate specific components of these pathways, transcriptional and epigenetic programs, cytoskeletal remodeling, and downstream cellular responses. *LASSIE* is shown as an example of an ncRNA involved in the mechanosensory machinery through its interaction with the VE-cadherin–cytoskeleton axis.Other examples of ncRNAs are reported in the red boxes, while blue boxes indicate protein effectors or axis, and purple/pink boxes indicate specific transcription factors. Solid arrows and inhibitory lines indicate positive and negative regulatory interactions, respectively, whereas dashed arrows indicate indirect or incompletely characterized mechanisms. Together, these pathways contribute to cardiovascular responses and pathological remodeling, including hypertrophy, atherosclerosis, valvular disease, and cardiac fibrosis.

The term “mechanotransduction” indicates the ability of the cells to transduce mechanical stimuli deriving from the environment (e.g., blood flow, matrix stiffness) into biological processes such as cell proliferation and differentiation (Figure 3) [72]. External cues propagate through the extracellular matrix (ECM) and the adjacent cells to activate membrane sensors that mechanically and biochemically transmit these forces [71]. In turn, downstream signaling pathways induce remodeling of cell and nuclear architecture, ultimately altering gene expression and cell phenotype [73]. Compression and traction forces, for instance, trigger the formation of nuclear pores and affect the trafficking of transcription factors [74,75]. Moreover, they can act at the epigenetic level by shaping chromatin topology and modulating the accessibility of the transcriptional machinery to silent heterochromatin [76], as well as by driving gene activation (e.g., histone acetylation and phosphorylation) or repression on specific targets (e.g., DNA methylation) [77,78]. These epigenetic changes possibly propagate with cell divisions, thus setting a cellular “memory” that affects lineage phenotype in the long term and allows cells to adapt to the changing biomechanical environment [79].

Many mechanosensing pathways start at the level of focal adhesions [80]. Here, transmembrane adhesion receptors (e.g., integrins, syndecans) sense external cues and transmit them to structural protein complexes bridging the ECM with the inner cytoskeleton. Integrins are highly characterized in the context of cardiac pathophysiology. Different integrin subunits, for instance, contribute to myofibroblast pro-fibrotic activation: the β1 subunit, contained in collagen and fibronectin receptors, is important for stiffness sensing and profibrotic signaling; the β2 subunit activates collagen remodeling through matrix metalloproteinases; the β3 subunit mediates ECM adhesion in pressure-overload conditions [81]. In turn, integrins transmit forces to adaptor proteins like Focal Adhesion Kinase (FAK), activating RhoA GTPase and Rho Kinase (ROCK), which in turn promote the cytoskeletal rearrangement and the formation of contractile stress fibers of α-smooth muscle actin (αSMA) (Figure 3) [81]. Mechanosensing ion channels are other important mediators that bridge environmental stimuli with phenotype changes (Figure 3). Among these, notable examples are the non-selective cation channels such as transient receptor potential (TRP) channels and the Piezo1 channel. TRPC3, TRPC6, and TRPV4 are particularly sensitive to matrix stiffness, stretch, and osmotic pressure [82]. TRPV4, importantly, is a Ca2+ channel that, upon mechanical stress, activates RhoA/ROCK, shaping the cytoskeleton and promoting nuclear importation of pro-fibrotic transcription factors [83,84]. Piezo1, similarly, mediates mechanical signaling in cardiac fibroblasts through Ca2+, and likely represents the most famous example of mechanosensing membrane structure, since its discovery [85,86,87].

One of the most important mechanotransduction pathways with cardiovascular relevance is mediated by the transcriptional co-activators Yes-associated protein (YAP) and its paralogue TAZ, which are canonical downstream effectors of the Hippo pathway [88]. In cardiac cells, exposure to stimuli including matrix stiffness, strain, and cell density can regulate Hippo pathway activation [89], resulting in a signaling cascade involving several kinases and associated proteins [73,90]. During mechanical stress, this drives YAP/TAZ dephosphorylation in the cytoplasm. Concurrently, the alteration of nuclear architecture deriving from the force transduction on the cytoskeleton enables nuclear pore opening, through involvement of the LINC complex, and YAP/TAZ importation into the nucleus (Figure 3) [91,92,93]. A multitude of complex processes can be additionally responsible for YAP/TAZ stabilization and shuttling, with implications in metabolic, inflammatory, and structural responses [73,90]. In the nucleus, binding to members of the TEAD transcription factor family (e.g., TEAD2/4) allows YAP/TAZ to ultimately act on gene expression, altering cell proliferation, differentiation, and pro-fibrotic behavior (Figure 3) [73,94]. Important target genes of YAP/TAZ are *Ctgf*, *Cyr61*, and *Ankrd1*, which establish a profibrotic program inducing *Acta2* (encoding for αSMA) and collagen-related genes, among many others [73,94].

Interestingly, the YAP1 gene also gives rise to a non-coding transcript called *circYAP*, which modulates mechanotransduction by directly antagonizing the translation of *YAP* mRNA [95]. This ncRNA further contributes to regulating cell structure and function by binding to the cytoskeletal proteins Actin Gamma-1 (ACTG) and Tropomyosin 4 (TPM4), reducing actin polymerization and reducing cardiac remodeling upon MI [96].

The interplay between multiple pathways is responsible for the complex orchestration of the responses to mechanical stress in the cardiovascular context, which includes not only YAP/TAZ, but also other mechanotransduction pathways, driven for example by p38/MAPK, NFAT, or MRTF-A (Figure 3) [75,97]. Other signaling networks may additionally participate in this interplay, such as Transforming Growth Factor beta (TGFβ) and Wnt/β-catenin pathways [73,97].

The role of TGFβ intertwines with mechanosensing, particularly in a fibrotic and inflammatory context, in both cardiac fibroblasts and macrophages. The TGFβ1 isoform, typically secreted in the ECM in an inactivated form, is the main mediator of this signaling. Upon mechanical stress, integrins undergo structural changes that lead to pro-TGFβ1 cleavage, activation, and release to initiate signaling on TGFβ receptors [98]. Among many others, the canonical downstream effectors of TGFβ1 are the SMAD transcription factors, which ultimately get phosphorylated and imported into the nucleus, where they control the expression of genes encoding collagens, integrins, and matrix metalloproteinases (Figure 3) [99]. Active YAP/TAZ in the nucleus can additionally recruit SMAD2, 3, and 4, thus connecting the two pathways in response to mechanical stress [100,101,102]. β-catenin activation from the Frizzled receptor (binding to Wnt ligands), as well as TGFβ-induced SMAD7, leads to altered YAP/TAZ turnover in the cytoplasm, thus finely tuning the cytoplasmic/nuclear ratios of mechano-effectors according to the biochemical, and not only mechanical, composition of the microenvironment (Figure 3) [103].

The activation of these pathways in response to mechanical stress, in combination with other biochemical pro-fibrotic or pro-inflammatory stimuli, is particularly relevant in the context of progressive cardiac fibrosis, where the interruption of mechanotransduction signaling may represent a beneficial approach. Moreover, the biological outcomes of these pathways may vary in different cell types, making mechanosensing effectors possibly tissue- and cell-specific and yet to be completely elucidated. In this context, overlooked effectors such as non-coding RNAs may be new interesting objects of research.

In this review, we define *mechano-ncRNAs* as non-coding RNAs whose expression, processing, localization, or activity is altered by mechanical cues. Mechano-ncRNAs are therefore, in a broad sense, mechanoresponsive and can be further classified into three functional categories based on the role of the ncRNA in the mechanotransduction cascade. Importantly, as the role of a given ncRNA may be cell-, stimulus-, or context-dependent, the same transcript can fall into more than one definition.

*Mechanosensors* are molecules that contribute to detecting mechanical cues and drive the initial cellular response, e.g., through physical association with focal adhesions, the cytoskeleton, or the nuclear envelope. An example is *LASSIE*, a lncRNA that physically links nestin to VE-Cadherin in endothelial cells, connecting adherens junctions to the cytoskeleton and driving the intracellular response to shear stress [104]. However, as mechanosensor ncRNAs are rare in the literature, we focus mostly on the remaining classes. *Mechanotransducers* are regulated by mechanosensitive pathways (e.g., YAP/TAZ, integrin/FAK, TGFβ), and, in turn, change the activity of those pathways or relay the signal to downstream components, often through feedback or feedforward loops. *MiR-148a-3p*, described later, falls into this category: modulated by cyclic stretch, in turn, it regulates the downstream activation of the inflammatory NF-kB pathway in valve interstitial cells [105]. A third class, *mechanoeffectors*, are ncRNAs acting downstream of the signaling pathway, directly affecting the phenotype in response to mechanical cues and without a demonstrated effect on the signaling cascade that regulates them. Because the absence of feedback may reflect missing evidence, this classification refers to the current experimental evidence. *MiR-21* is an example of a stiffness-modulated mechanoeffector that promotes a profibrotic phenotype in cardiac fibroblasts [106].

Assignment to different categories requires causal evidence (e.g., loss- or gain-of-function validation) in a relevant model, while expression changes alone support only a general mechanoresponsive behavior.

Transcripts that are merely altered in diseases with a mechanical component, without evidence of a direct link to a specific stimulus, are not considered mechano-ncRNAs but rather *mechanically associated*. Several non-coding transcripts have been found broadly involved with the modulation of mechanotransduction pathways in the cardiac context, with implications for cardiovascular function, but have not been recognized as responsive to a specific stimulus (Table 1).

Examples of these are *miR-133* and *miR-30*, which appear to negatively regulate the expression of connective tissue growth factor (*CTGF*) [107], a canonical target of the YAP/TAZ pathway and an important effector in the process of fibrosis. The forced overexpression of *miR-133* and *miR-30* was found to induce a decrease in *CTGF* levels and collagen production in cardiac hypertrophy rodent models [107]. Given the implication of *CTGF* in YAP/TAZ mechanotransduction, this represents an interesting example of how intervening at the level of non-coding RNAs may regulate the mechanically induced fibrosis in the heart. *MiR-34*, instead, is known in the cardiac context to modulate the expression levels of transcripts encoding for vinculin, Notch1, and Bcl6, thus affecting mechanotransduction directed by focal adhesions [108]. In the context of CVDs, silencing *miR-34* in mouse models of transverse aortic constriction (TAC) and myocardial infarction (MI), characterized by altered mechanical conditions, was found to prevent adverse remodeling and to restore cardiac function [108].

Other examples of mechanically associated ncRNAs are *miR-29* and *miR-33*, whose levels were found to be altered in the murine and human myocardium upon infarction, dilated cardiomyopathy, and pressure overload [109,110]. Mechanistically, *miR-29* inhibits a plethora of mRNAs involved in ECM remodeling [109], thus regulating the pathological fibrotic deposition responsible for scar formation and increased stiffness. *MiR-33*, instead, was shown specifically to modulate ECM remodeling and fibrotic responses by inducing the matrix metalloproteinase 16 (MMP16) and the MAPK pathway, as well as by preserving lipid raft cholesterol content [110,111].

Another notable example of a ncRNA that acts as a mechanically associated transcript but is not directly linked to specific biophysical stimuli is represented by the circRNA *circHelz*, which was found upregulated in fibrotic murine hearts [112]. Mechanistically, *circHelz* is regulated by the general stress-responsive NFATC2 factor. In conditions of cellular stress, its function is to facilitate the nuclear translocation of YAP1, ultimately enhancing cardiac fibrosis in vitro and in vivo [112]. In parallel, *circHelz* drives a parallel pathological response in cardiomyocytes, suppressing *miR-133-3p*, resulting in pyroptosis [113]. *CircNfix*, similarly, was found to modulate the overactivated Hippo/YAP pathway in cardiomyocytes and negatively regulate cardiac regeneration in MI preclinical models [114]. In particular, *circNfix* binds to RNA-binding proteins that affect the degradation of Y-box binding protein 1 (Ybx1) [114], a modulator of *YAP1* transcript [115] and an interactor of YAP/TAZ in the nucleus [116]; therefore, potential approaches aiming at silencing *circNfix* could be innovative ways to selectively modulate YAP activity in the heart.

Although several ncRNAs can be found implicated in cardiac mechanotransduction, their regulatory functions often closely depend on the nature and magnitude of the mechanical stimulus. In several cases, it is only possible to postulate a role for ncRNAs in connecting biochemical stimuli with a phenotypic change in CVDs, as a large portion of the literature focuses more on correlative disease-driven studies rather than molecular studies. Since the exact mechanisms driving the ncRNAs’ responses to mechanical alterations are sometimes yet to be fully deciphered in the CVD context, claims on their role as bona fide mechanosensors, -transducers, or -effectors should be restricted to the provided evidence and context.

In the next sections, we will focus on examining individual biomechanical cues, which provide a more comprehensive understanding of the mechanisms through which mechanoresponsive ncRNAs contribute to cardiovascular homeostasis and disease.

### 3.2. Mechano-ncRNAs Responsive to Matrix Stiffness

Among cardiovascular diseases, cardiac fibrosis is particularly linked to mechanical stress, and several studies have proven the involvement of non-coding RNAs in the pathology as regulators or biomarkers. In the heart, cardiac fibroblasts (CFs), one of the main producers of the ECM, play an essential homeostatic role in structural and mechanical cardiac integrity [70]. They are activated by inflammatory, ischemic, and metabolic insults, thereby differentiating into myofibroblasts and promoting cardiac fibrosis, which leads to scar formation [73]. This maladaptive remodeling process is characterized by the presence of several simultaneous mechanical triggers that maintain CFs in a chronically active state: increased stiffness due to scar formation or excessive interstitial ECM deposition, increased strain due to reduced myocardial compliance in systole and/or diastole, and increased hydrostatic pressure in the regions exposed to blood flow [80].

Recent data published by our group have demonstrated that human cardiac fibroblasts respond to variations in tissue mechanics by activating the YAP/TAZ transcriptional complex and that pharmacological inhibition of this pathway is sufficient to reduce cardiac fibrosis in vivo [117,118]. The physiological stiffness of the heart in systole, typically defined through a parameter called Young’s elastic modulus, measured in Pascals (Pa), reaches 8 kPa on average [80,119]. In this relatively soft condition, the nucleus is not subjected to physical alteration, and the active transport of proteins like YAP/TAZ across the nuclear membrane is prevented [91]. In pathological conditions (e.g., upon MI or chronic hypertrophy), myocardial stiffness can witness up to a tenfold increase, reaching 100 kPa on average [120,121]. In the case of the YAP/TAZ pathway, the stiffer extracellular matrix may activate mechanotransduction through surface receptors like the angiotensin type 1 receptor (AT1R), and later through RhoA/ROCK and the cytoskeleton, opening nuclear pores and allowing the shuttling of the effector [91]. Forces acting on the cytoskeleton can be transmitted to the nuclear envelope and to the LINC complex, affecting lamin-associated chromatin organization and gene expression [93].

Pathological stiffness affects cardiac fibroblasts also by negatively acting on fibrosis repressors, which are often involved in metabolic regulation [122]. The engagement of the mechanosensing apparatus in CFs, driven by the progressive stiffening of the matrix, could initiate a transcriptional gene network that is permissive for chronic scarification in the infarcted heart and is responsible for balancing pro- and anti-fibrotic pathways with involvement of non-coding RNAs. From this perspective, it is possible that a positive feedback loop exists, in which fibrosis increases stiffness, stiffness induces mechano-ncRNAs, which may further promote fibrosis (unless specifically involved in other regulatory roles) (Table 2).

A network of miRNAs was found to be involved in the control of the biomechanical homeostasis of cells and tissues, with a conservation across cell types and species [129]. The transcriptional levels of these miRNAs appear modulated by substrate stiffness, with their main targets being among mRNAs encoding cytoskeletal, contractile, adhesion, and ECM proteins [129]. To support the claim that these miRNAs with transducer roles intervene in mechanotransduction, the inhibition of their mRNA target recognition was shown to compromise the in vitro and in vivo adaptation to stiffness, with an increase in cell contractility and YAP transcriptional activity even in cells growing on soft matrices [129]. Therefore, the post-transcriptional regulation mediated by mechanoresponsive miRNAs seems to be necessary to maintain biomechanical cell homeostasis.

An example of a miRNA responsive to stiffness is represented by *miR-21*. A stiff substrate matrix upregulates its levels through the mechanosensitive transcription factor MRTF-A, as shown in human mesenchymal stem cells (MSCs) [123]. *MiR-21* is known to be deregulated in a multitude of CVDs such as heart failure, MI, or proliferative vascular disease [130,131] that are subjected to altered stiffness conditions, primarily, and additionally to strain and shear stress. In cardiac fibroblasts and rodent MI models, this miRNA was shown to promote myofibroblast profibrotic conversion by targeting Jagged1, ultimately offering a potential mechanism to target for therapeutic intervention in cardiac fibrosis [106].

Regarding lncRNAs, some of them have only been found to be correlated with profibrotic processes and cardiac remodeling (e.g., *MIAT*) [132], and their role has not been directly linked to specific conditions of stiffness through dedicated studies. However, other examples in the context of high stiffness-CVDs may be found in the literature. The mechanoresponsive lncRNA *CYTOR*, for instance, was found to modulate gene expression in mesenchymal stromal cells under changing 3D stiffness conditions [124]; moreover, an independent study showed that *CYTOR* modulates the *miR-155*/NF-kB axis in murine in vitro and in vivo models of cardiac hypertrophy [125]. Although the regulation of *CYTOR* by matrix stiffness remains to be validated in cardiovascular cell types, these observations suggest a potential mechanically altered molecular axis to further investigate in cardiovascular diseases. Similarly, *NEAT1*, which was first characterized as stiffness-responsive in multiple cancer contexts [133,134], was also found to significantly impact cardiovascular function in cardiomyocytes, regulating apoptosis in response to hypoxia [126], and in cardiac fibroblasts, inducing fibrosis by repression of SMAD7 [127].

Vascular extracellular matrix stiffening, which is influenced by hydrostatic pressure and altered blood flow, is a risk factor predisposing to aortic and coronary artery disease. A notable example of a mechano-ncRNA involved in its pathogenesis, as well as implied in other CVDs, and working as a stiffness-responsive mechanoeffector, is the lncRNA *MALAT1*. *MALAT1* is downregulated by matrix stiffness in human vSMCs, where it affects cell migration and proliferation in vitro, and neointima formation in vivo, in response to ECM pathological accumulation [128]. Still, the upstream mechanotransduction pathway responsible for the stiffness-dependent reduction of *MALAT1* remains unresolved.

### 3.3. Mechano-ncRNAs Responsive to Cyclical Strain and Pressure Overload

Another important source of mechanical stress in the cardiovascular context is represented by strain, that is, the periodic deformation of cardiac cells and tissue generated by the constant contraction-relaxation cycles of the beating heart. Strain and pressure overload are distinct stimuli, the latter being a sustained increase in the hemodynamic load of the ventricular wall. In this section, cyclic strain and pressure overload will be discussed together because of their mechanical link: pressure overload increases wall stress and thereby the amplitude of cyclic strain experienced by cardiac cells, which sense both through partly overlapping mechanosensors [135,136,137].

In conditions including cardiac hypertrophy, hypertension, and post-ischemic damage, the strength of contraction required to efficiently pump the blood into circulation is higher due to the pressure overload of the tissue; therefore, the tension and compression forces perceived by cardiac cells are also more intense [135,138]. In the case of myocardial infarction, the border zone (i.e., the area immediately adjacent to the ischemic zone) is characterized by a high migration rate of cardiac fibroblasts, together with matrix metalloproteinase expression and collagen denaturation, leading to strong ECM remodeling [139,140]. This effect, combined with the contraction of the remaining cardiomyocytes at the border zone, drives the establishment of chronic mechanical stress on the tissue and on the newly recruited cardiac fibroblasts, further exacerbating the pathological scenario [141].

The cellular effect of cyclic strain has been documented both in vitro and in vivo. In 3D-cultured cardiomyocytes, stretching forces up to 10%, combined with their endogenous contraction, influence sarcomeric protein accumulation and turnover in accordance with the direction of the applied forces [142]. Indeed, transverse forces and systolic stretch are known to induce different rearrangements and signaling pathways compared to longitudinal forces and diastolic stretch, thus accounting for an adaptability of cardiomyocytes to the specific mechanical stimulation during development and disease [99,142]. In rat ventricular fibroblasts, exposure to 9% strain with continuous cyclical stretching at 0.33 Hz induces upregulation of collagens I and III, matrix metalloproteinase 2 (MMP-2), and tissue inhibitor of MMP2 (TIMP2), which initiate a profibrotic response to the mechanical stress [143]. This response participates in crosstalk with macrophages, which, in the profibrotic environment of hyper-stretched fibroblasts, secrete TGFβ and pro-inflammatory cytokines [144]. Similar to stiffness, mechanical stress due to stretching forces activates the previously described signaling pathways, such as YAP/TAZ [145], with comparable outcomes.

Several mechano-ncRNAs induced by mechanical strain have been implicated in the transition from adaptive hypertrophy to pathological cardiovascular remodeling (Table 3). However, despite their functional relevance, the processes governing their upstream induction or repression in response to mechanical strain still have to be fully elucidated.

Cardiac hypertrophy is a direct consequence of sustained pressure overload: starting as an initial physiological response, when the load (and associated strain) persists, it becomes maladaptive and progresses to heart failure [137]. In particular, it leads to changes in the mechanical composition of the myocardium and to hypertrophic growth of cardiomyocytes, with an overall thickening of the heart [137]. Hypertrophy is not exclusively triggered by mechanical strain; therefore, ncRNAs altered in pressure overload/hypertrophy models should not automatically be considered responsive to strain.

A signature of mechano-miRNAs directly responsive to strain has been identified in cardiac myocytes, in which the *Let-7* family of miRNAs was particularly dysregulated [150]. Interestingly, *Let-7* miRNAs are involved in pathogenic mechanisms of CVDs, including ventricular hypertrophy [146]. Mechanistically, *let-7a*, in particular, was shown to have anti-hypertrophic activity by directly affecting calmodulin (CaM) expression in neonatal rat ventricular myocytes [147]. Although the functional role in linking direct strain to this phenotype remains to be validated, *let-7a* is a potential mechanoeffector candidate, according to our definition.

A well-characterized example under controlled strain is *miR-208a*, a mechanotransducer. In rat cardiac myoblasts subjected to cyclic stretch, *miR-208a* expression increases through TGFβ1 signaling, which is relayed downstream by promoting the expression of endoglin and collagen I. AntagomiRs or inhibition of SMAD2/3 attenuate this response [148]. This mechanistic effect was also shown in vivo, in a rat model of volume overload, where *miR-208a* acts as a driver of myocardial fibrosis [149].

A large part of the literature describes the role of ncRNAs as biomarkers or functional mediators of the disease; however, in many cases the direct responses to cardiac strain have not been demonstrated. Therefore, we consider these as additional mechanically associated examples, such as the ncRNAs in Table 1. In particular, *miR-132* was found to promote maladaptive remodeling and hypertrophy, and its suppression with the inhibitor CDR132L improved myocardial strain and function in animal models [151,152]. The same treatment in clinical trials did not reach primary efficacy endpoints, although exploratory analyses suggested a possible benefit in patients with advanced remodeling [153]. Among lncRNAs mechanically associated with high-strain CVDs, *CHAST* and *Meg3* are dysregulated in pressure overload models, where they drive hypertrophic and maladaptive remodeling responses through different mechanisms in various cardiac cell types [145,154,155].

### 3.4. Mechano-ncRNAs Responsive to Shear Stress

Other important biomechanical forces derive from shear and hydrostatic stress, which impact the vascular system at the level of endothelial cells (ECs) and vascular smooth muscle cells (vSMCs) [156,157]. The hemodynamics of the circulating blood determine the formation of water flow within tissues and cells in different directions, which macroscopically create tension and compressive strain (hydrostatic stress) on vascular cells, which can promote pathological processes [99]. The endothelium, indispensable for regulating blood pressure and vascular permeability, can typically sense such biomechanical triggers and consequently secrete specific factors to modulate the vascular tone through vSMCs [158].

Shear stress, generally determined by the biophysical frictional forces exerted by the blood on the vascular cells, can be laminar (LSS), more involved in vascular homeostasis, or oscillatory (OSS), more pathological and dependent on pressure alterations. In atherosclerosis, disturbed OSS at arterial bifurcations promotes endothelial dysfunction and atherosclerosis [158]. In arterial hypertension, increased vascular wall stress from sustained pressure overload activates mechanotransduction pathways in ECs and vSMCs, leading to vascular remodeling and increased arterial stiffness, with an impact on general cardiovascular performance [159]. In a similar way, shear stress may affect the compaction of valve interstitial cells (VICs) during early development, and later drive the formation of cardiac valves and their pathological calcification [160].

Shear stress regulates a plethora of ncRNAs, which in turn determine whether vascular cells acquire a protective or inflammatory phenotype. Notable examples of the best-validated ncRNAs are classified here according to their role in the mechanotransduction cascade (Table 4).

Many miRNAs were identified as mechano-ncRNAs because their biogenesis is directly affected by laminar flow or oscillatory flow.

Flow directly controls the processing of *miR-93* and *miR-484*. Under pulsatile flow (where pressure and velocity of flow change over time), AMPK-dependent phosphorylation of nucleolin, a miRNA-processing shuttle protein, prevents the maturation of both miRNAs. Under OSS, this phosphorylation is lost, allowing nucleolin-dependent maturation and shuttling of the transcripts to the cytoplasm. In this compartment, *miR-93* and *miR-484* were found to repress the targets *Krüppel-like factor 2* (*KLF2*) and *eNOS*, leading to endothelial dysfunction [161]. In the same study, the levels of both miRNAs were also found to be elevated in sera from human patients with coronary artery disease (CAD), suggesting a possible biomarker role [161]. As KLF2 is a central flow-responsive transcription factor, these miRNAs act as mechanotransducers, relaying the flow pattern to the KLF2/eNOS program. Similarly, the murine-specific *miR-712* is upregulated by disturbed flow: mechanistically, OSS initially reduces the levels of XRN1, an exonuclease responsible for degrading *miR-712*, leading to its accumulation [162]. *miR-712* in turn represses the transcription of tissue inhibitor of metalloproteinase 3 (TIMP3) while prompting the expression of matrix metalloproteinases (MMPs), favoring endothelial inflammation; in vivo, its silencing prevents atherosclerosis in mice [162]. Moreover, the human *miR-205*, which shares its seed sequence, was shown to conserve the flow-responsive behavior and function, targeting TIMP3 [162]. Given this evidence, *miR-712* can be defined as a mechanoeffector in mice.

In endothelial cells, in response to LSS, *miR-21* has a positive impact, modulating apoptosis and eNOS activity, improving endothelial cell function [131]. In this context, *miR-21* acts as a shear-stress-responsive mechanoeffector. In addition, its peripheral blood levels have also been negatively associated with arterial stiffness [130], and it has been associated with cardiac fibroblast activation [106]. Given the highly context-dependent role of *miR-21*, careful consideration should be taken for clinical translation of *miR-21* modulators as anti-atherosclerotic or anti-fibrotic treatments.

Among lncRNAs, the two lncRNAs *LEENE* and *LASSIE*, both derived from the gene locus *LINC00520*, are known to be LSS-responsive and involved in the regulation of endothelial function. As previously mentioned, *LASSIE* is associated with junctional proteins and nestin, linking adherens junctions to the cytoskeleton; its silencing impairs cell alignment in the direction of the flow [104]. We classified it as a mechanosensor ncRNA in this context. *LEENE*, a nuclear enhancer-associated lncRNA, promotes instead eNOS transcription by recruiting RNA polymerase II to its promoter, thus working as a mechanoeffector [163]. Upstream, LSS directly drives the stress-sensitive KLF2/4 factors to upregulate both *LEENE/LASSIE*, thus modulating endothelial function homeostasis through two independent mechanisms [104,163]. Another LSS-dependent mechano-ncRNA involved in the KLF4 pathway is *AF131217.1*. This lncRNA also has an anti-inflammatory role in ECs in response to LSS. To exert its function, it sponges *miR-128-3p*, thereby regulating its target KLF4 and, indirectly, the expression of eNOS and endothelial adhesion molecules like VCAM and ICAM [163]. As KLF4 in ECs is considered an important shear stress-induced transcription factor [167], this study suggests that *AF131217.1* acts as a mechanotransducer. CircRNAs also participate in flow responses. For example, altered blood flow directly affects the expression levels of *circRNA-LONP2* in endothelial cells, which sponges miR-200a-3p, promoting Keap1/YAP1/EZH2 and blocking NFE2L2/HMOX1 signaling. Ultimately, this leads to endothelial oxidative stress and inflammation, and drives atherosclerosis [165]. *CircRNA-CZFP292*, instead, is required for laminar flow-induced cytoskeletal reorganization: it binds the protein syndesmos (SDOS), allowing endothelial cell alignment according to flow and focal adhesion organization in vitro [166]. In vivo, the genetic depletion of *circRNA-CZFP292* disrupts flow alignment of the aortic endothelium, hampering the mechanical response to laminar shear stress [166].

All the previously mentioned examples shed light on the putative link between mechanosensing, ncRNAs, and CVDs, but increasing research still must provide more insights and validations into their specific mechanistic roles and their involvement with the biomechanical response. Transcriptomic sequencing approaches may provide additional insights by identifying candidate ncRNAs at the crossroads of mechanosensing and CVDs. For instance, Sharma et al. (2020) [168] identified by bulk RNA-seq several stiffness-responsive lncRNAs (e.g., *AK050884*, *ENSMUST00000134701*, *TCONS_00000072*, *ENSMUST00000141797*) linked to mechanosensory pathways involving TGFβ- and TRPV4-mediated signaling. Although this investigation was not conducted on cardiac cells, similar studies may provide lists of interesting candidates for further research.

## 4. Approaches for Discovery and Validation of Mechanosensitive ncRNA

**Figure 4:**
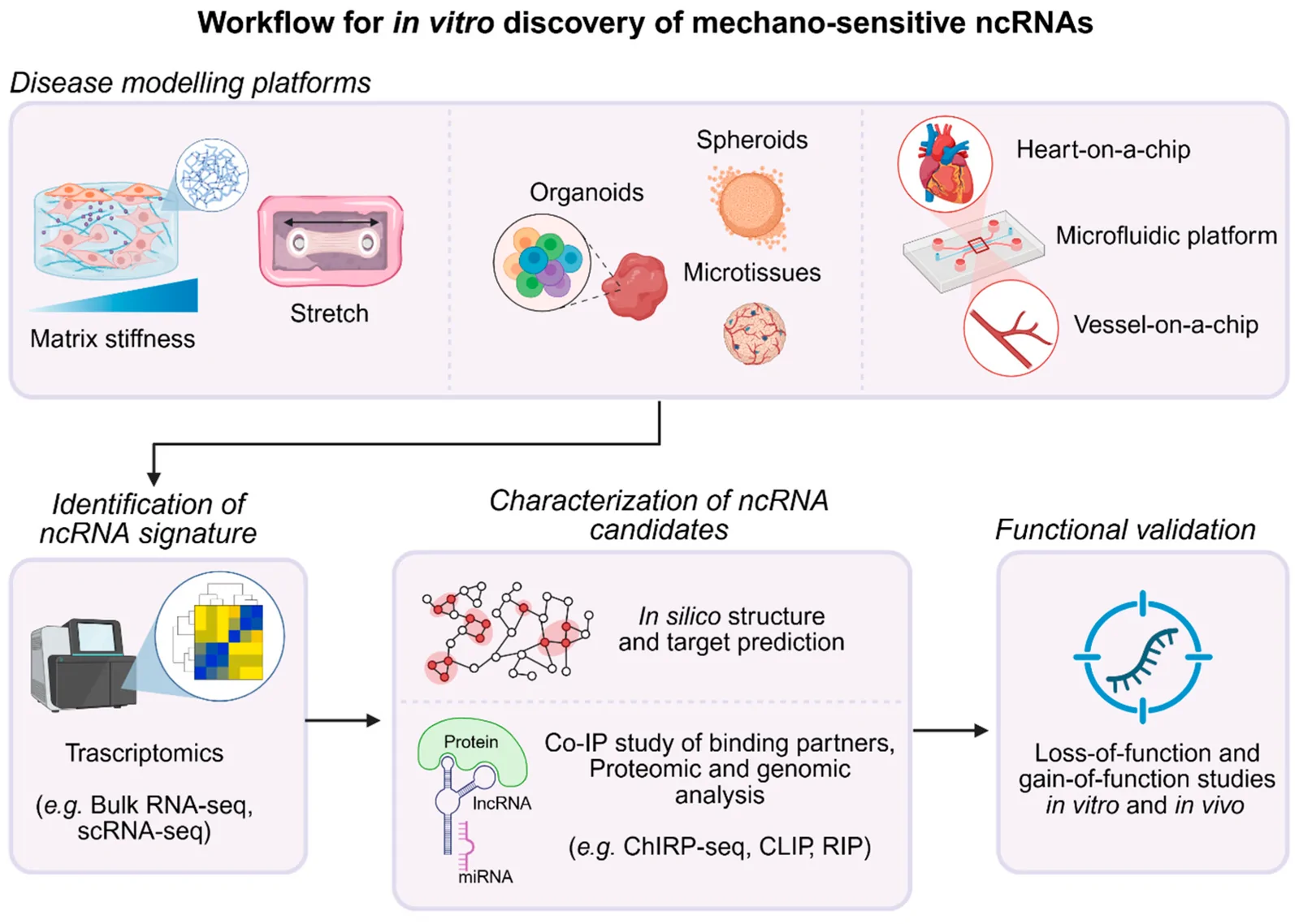
Workflow for in vitro discovery of mechano-sensitive ncRNAs. Disease modeling platforms can be used to simulate physiopathological conditions in 2D or 3D systems, to investigate the effect of biomechanical stimuli and discover new mechano-ncRNAs. Matrices of differential stiffness or cell/tissue stretchers can be used to apply stiffness- and strain-related mechanical forces; organoids, spheroids, and microtissues can be employed to best recreate the 3D architecture of the cardiovascular tissues; microfluidics platforms can be used to build hearts-on-a-chip or vessels-on-a-chip to study shear stress in vascularized systems. The biological effect of biomechanical cues can be analyzed with transcriptomic techniques to identify a signature of differentially expressed mechano-sensitive ncRNAs. The mechanism of action, targets, and genomic regulation of the ncRNA candidates can then be investigated through in silico predictions or in vitro immunoprecipitation assays coupled with other omics techniques. Finally, the candidates require validation of the biological function and clinical impact, evaluated by loss- and gain-of-function studies in vitro and in vivo.

In light of the evidence discussed above, ncRNAs can be viewed as intermediates, amplifiers, buffers, or memory elements linking mechanical stimuli to downstream transcriptional responses. These findings offer opportunities for the development of novel therapeutic and diagnostic strategies for cardiovascular diseases, either as standalone approaches or in combination with conventional therapies. However, fully exploiting their therapeutic and diagnostic potential will require the development of experimental and computational discovery platforms capable of systematically identifying mechanosensitive ncRNAs and their regulatory networks (Figure 4). In this context, techniques for ncRNA discovery and profiling range from approaches that simulate cardiovascular mechanical stress in vitro or in vivo to innovative, AI-based computational approaches, including multi-omics and target prediction analyses. Finally, validation of the identified ncRNA signatures in vivo or in patient samples enables correlation of the results obtained in bioengineered models with the clinical manifestations of CVD.

The discovery of ncRNA biomarkers requires careful consideration of several biological and technical factors that may influence their clinical relevance and reproducibility of detection. After briefly discussing biomarkers, this section reviews the main strategies for modeling mechano-related CVDs and characterizes mechanosensitive ncRNA candidates for future clinical applications.

### 4.1. Considerations on Biomarkers

The detection of circulating ncRNAs could improve the prediction of disease onset, progression, and clinical outcome. Several biomolecules, including proteins, enzymes, hormones, and coding and non-coding RNAs, have been associated with cardiovascular diseases [169,170,171]. Protein biomarkers such as cardiac troponin T (cTnT) and natriuretic peptides remain the clinical gold standard: troponins are released upon myocardial injury, while natriuretic peptides are released in response to myocardial wall stress, and both can be measured with highly sensitive assays [171]. However, individual biomarkers often lack sufficient specificity to accurately define disease phenotypes, highlighting the need for complementary biomarker panels. Since only a limited number of proteins are truly cardiac-specific, circulating ncRNAs, which can show cell- and tissue-restricted expression, represent candidate companion biomarkers [170]. An example is lncRNA *LIPCAR*, which has been identified as a prognostic biomarker in heart failure associated with cardiac remodeling, although it is not exclusively of cardiac origin [172].

Important biomarkers for cardiovascular diseases can be found among several types of ncRNAs, both intracellularly and extracellularly, where they can be transported within extracellular vesicles [3,173]. Extracellular ncRNAs are particularly attractive due to their accessibility through minimally invasive sampling of biofluids, such as plasma or serum. Therefore, investigating the non-coding cardiovascular secretome in response to biomechanical stimulation may represent a valuable strategy for identifying and validating novel mechano-regulated biomarker signatures. An additional advantage of ncRNAs is their rapid release during the early phases of disease. Cardiac-enriched miRNAs, for instance, can be detected as early as one hour after the onset of ischemia, preceding many protein biomarkers and potentially enabling earlier therapeutic intervention in myocardial infarction [174].

Nevertheless, the interpretation of ncRNA biomarkers requires caution. The tissue of origin of a circulating ncRNA is often uncertain, and measured levels can be influenced by pre-analytical variables such as sample type, processing, hemolysis, and storage [175]. Additionally, their diagnostic or predictive role may be biased by inaccurate normalization strategies and by comorbidities [175]. Therefore, although ncRNA biomarkers hold considerable promise, their clinical implementation will require validation in large patient cohorts together with optimized and standardized protocols, as well as proper normalization strategies and automated detection platforms, to ensure reproducibility [171].

The validation of circulating ncRNAs generally relies on quantitative Reverse Transcription PCR (RT-qPCR), an inherently specific technique that, although preferred for the generally short times of analysis, is limited by costs and technical reproducibility, due to the absence of a consensus on protocols and standardization controls [175]. Another caveat is that many cardiac-derived ncRNAs are poorly abundant and may escape detection by conventional RT-qPCR assays, unless pre-amplification steps are introduced or more sensitive techniques are employed, such as digital droplet PCR (ddPCR) [175].

### 4.2. Mechanobiology Platforms

Mechanobiology research tools that can reliably apply specific or combinatorial types of mechanical stress may facilitate the identification and screening of novel candidate targets (Figure 4).

Three-dimensional matrices that mimic the native myocardial or vascular extracellular matrix (ECM) are widely used to investigate cardiovascular mechanobiology. For instance, stiffness gradient polyacrylamide gels (2–33 kPa) have been employed to model healthy and diseased myocardial tissue and to study stiffness-dependent mechanotransduction in embryonic and neonatal rat cardiomyocytes [176]. Since tissue stiffness changes dynamically in vivo, biomaterials with tunable mechanical properties have been developed to provide temporal control of matrix rigidity through approaches such as biochemical crosslinking or modulation of magnetic and conductive behavior (Figure 4) [177,178,179]. These platforms, particularly when combined with omics analyses, enable the identification of mechanosensitive molecular pathways. For instance, models of tunable stiffness coupled with RNA sequencing of in vitro and in vivo samples recently identified a set of 98 mechanically responsive genes in cardiac fibroblasts, including *THBS2* and *PCSK6* [180]. A similar strategy applied for the discovery of ncRNAs could define mechanoresponsive ncRNA signatures, providing novel therapeutic targets for mechanically driven cardiovascular diseases beyond canonical protein targets.

Mechanical stretch platforms provide complementary approaches to study cellular responses to strain [181]. Both two-dimensional (2D) and three-dimensional (3D) systems enable the application of controlled static or dynamic, uniaxial or multiaxial deformation, allowing the characterization of strain-responsive molecular changes in cardiac and vascular cells, including ncRNAs (Figure 4) [182,183]. The development of high-throughput devices, such as 192-well stretch platforms, further expands the possibility of combining mechanical stimulation with screening and multiomic approaches for the identification of mechano-regulated ncRNAs [184].

Although traditional 2D cultures often fail to reproduce the structural complexity of cardiovascular tissues, more advanced 3D models, including organoids, spheroids, microtissues, and organs-on-a-chip (OOC), provide improved control of tissue organization and mechanical stimulation (Figure 4). Heart-on-a-chip and vasculature-on-a-chip platforms, for example, can be integrated with biochemical, electrical, and mechanical cues through approaches such as perfusion, pneumatic strain, and flow modulation [185,186,187], potentially enabling the modeling of pathological environments associated with myocardial infarction, fibrosis, or hypertension. Due to the limited vascularization and cellular organization of many 3D systems, microfluidic approaches have emerged as particularly useful for studying flow-dependent mechanisms. By controlling fluid dynamics within microchannels and incorporating multiple cell types, vessel-on-a-chip models can reproduce relevant aspects of vascular architecture and function, including systems based on human induced pluripotent stem cell (hiPSCs)-derived ECs, vSMCs, and mural cells [186]. Together, organs-on-a-chip and microfluidic platforms provide versatile tools to investigate mechanically regulated cell interactions, barrier function, disease progression, and cardiovascular development, while enabling the identification of ncRNAs responsive to defined mechanical conditions.

Although several other innovative platforms are continuously emerging, their detailed discussion is beyond the scope of this review. Nevertheless, a major challenge remains the limited scalability of current systems for mechanical stimuli, which often require the integration of additional screening platforms for subsequent molecular and functional analyses. Moreover, most in vitro platforms reproduce only selected components of the cardiac mechanical environment and therefore may not fully recapitulate the complex, multiaxial, and temporally dynamic combination of tensile, compressive, stiffness-induced, and shear forces experienced by cardiovascular cells in vivo.

### 4.3. Transcriptomic Approaches

High-throughput transcriptomics is the main strategy to identify ncRNA signatures in mechanically stimulated cells. RNA sequencing (RNAseq) can detect a broad range of coding and non-coding transcripts, according to the library preparation [188], facilitating the discovery of ncRNAs associated with cardiovascular diseases (Figure 4) [188,189,190].

Conventional poly(A)-selected RNAseq, the most widely used protocol for bulk and single-cell datasets, mainly captures polyadenylated transcripts (e.g., mRNAs, lncRNAs), but under-represents small RNAs (e.g., miRNAs), non-polyadenylated lncRNAs, circRNAs, and, in general, low-abundance transcripts [191]. Thus, a specific approach should be chosen according to the ncRNA class of interest, due to the distinct properties, size, localization, and generally low abundance of ncRNAs.

Total RNAseq after ribosomal RNA depletion is better suited to lncRNAs and circRNAs, as it preserves both polyadenylated and non-polyadenylated RNAs [192]. Dedicated small RNAseq protocols are required for miRNAs and other small ncRNAs, with adapters and size selection optimized for short ncRNAs [192]. Long-read sequencing, instead, can further resolve lncRNAs and circRNAs by obtaining information on full-length transcripts and their specific isoforms [193,194].

While bulk RNAseq shows average changes across mixed cell populations, single-cell RNAseq approaches can identify mechanoresponsive signatures in specific subpopulations. Even if more resolutive, poly(A)-based single-cell methods detect poorly expressed ncRNAs inefficiently; however, technical advancements in single-cell total RNAseq are extending coverage to coding and non-coding transcripts [189,195].

Whatever the platform, ncRNA discovery requires appropriate design and analysis, including time-matched, unstimulated controls, and biological replicates; normalization should be suited to the RNA class (e.g., with spike-in controls for small RNAs) [188,190].

Following discovery, candidate ncRNAs require functional annotation to define their genomic organization, regulation, biogenesis, and biological relevance. Overall, transcriptomic approaches allow the identification of ncRNA alterations across tissues, cell types, and pathological conditions, contributing to the understanding of their roles in cardiovascular biology and disease [188,189]. Examples of CVD-implicated ncRNAs discovered through these techniques are summarized in [196].

### 4.4. Functional Validation

Bioinformatic tools, such as ENCORI/starBase [197], miRbase [198], miRDB [199], AnnoLnc [200], miRanda [201], DIANA [202], and NONCODE [203], enable the prediction and annotation of ncRNAs, including their potential interactions with target miRNAs, mRNAs, lncRNAs, circRNAs, and RNA-binding proteins (Figure 4). Additional tools, such as Forna [204] and RhoFold+ [205], allow the prediction of ncRNA secondary structures, which may influence their binding properties and biological functions.

Several experimental approaches allow the characterization of ncRNA-mediated epigenetic regulation and molecular interactions (Figure 4). For example, Chromatin isolation by RNA Purification followed by sequencing (ChIRP-seq) identifies genomic regions associated with specific ncRNAs, particularly lncRNAs, by capturing RNA-chromatin complexes and integrating the results with genomic, transcriptomic, or epigenomic datasets. Other co-precipitation (pulldown) techniques, such as Cross-Linking Immuno-Precipitation (CLIP) and RNA Immuno-Precipitation (RIP), characterize the interactions between ncRNAs and downstream protein effectors [206,207]. Combined with sequencing approaches, these methods enable the definition of ncRNA interactomes and the investigation of their mechanisms of action.

The integration of transcriptomics with genomics, epigenomics, and proteomics through multi-omic approaches may provide a comprehensive view of the identity, regulation, and function of ncRNAs of interest. Several studies have applied this integration effectively to discover disease-associated ncRNAs and their mechanisms, combining techniques such as RNA sequencing, proteomics, metabolomics, and computational analyses [208]. However, the large amount of data generated requires robust quality control, standardization, and advanced analytical approaches. Machine learning and AI-based algorithms can support this process, extracting relevant patterns from complex datasets and identifying ncRNA-based diagnostic or prognostic signatures with potential applications in patient stratification [209,210,211].

Importantly, omics data can be predictive but are susceptible to library and mapping biases [188]; therefore, changes in candidate ncRNAs should be confirmed with practical methods, including RT-qPCR. In this case, expression levels should be normalized against multiple stably expressed reference genes or spike-ins [188] whose stability has been verified under the specific mechanical stimulus. Where relevant, other techniques should be used to confirm transcript size and subcellular localization, such as northern blotting or RNA fluorescence in situ hybridization. For circRNAs, importantly, appropriate validation should include different assays: the amplification of the back-splice junction with divergent primers, confirming the amplicon by further sequencing; and the RNAse R resistance test, to verify that the transcript is truly circularized [194].

Finally, to demonstrate the actual biological impact of candidate mechanosensitive ncRNAs, dedicated in vitro or in vivo experimental setups should be designed. For this purpose, loss-of-function (e.g., siRNAs, antisense oligonucleotides, gapmers, CRISPRi, or CRISPR-CAS9 mediated deletion) and gain-of-function (e.g., plasmids, lentivirus, AAVs, CRISPRa, etc.) approaches allow a first validation of ncRNA activity. The choice of strategy should take into account the ncRNA class, its biology, and subcellular localization. For instance, for lncRNAs, subcellular localization may help to design a more effective silencing strategy, as siRNAs are preferred for silencing cytoplasmic lncRNAs, while ASOs are highly suitable for silencing nuclear lncRNAs [212].

This can then be followed by specific biological assays to assess their role as novel biomarkers or effectors of a pathological condition [212]. Once validated, mechano-ncRNAs could be leveraged by personalized medicine approaches to design tailored treatments with RNA therapeutics for CVDs characterized by chronic mechanical overload, potentially improving therapeutic specificity by targeting mechanotransduction.

## 5. Targeted Therapeutic Modulation of Cardiac ncRNAs

Given the central role of biomechanics in cardiovascular disease, targeting mechanosensing pathways emerges as a promising therapeutic strategy [213]. In this context, ncRNAs-based approaches may offer advantages over conventional drugs, as sequence-specific base pairing can enable selective modulation of disease-associated transcripts, potentially including otherwise “undruggable” targets [214]. Moreover, the role of ncRNAs is often context-dependent, with expression levels and mechanisms of action depending on tissue and cell type; such a unique window of activity may offer more specific targeting conditions with potentially reduced adverse effects [215]. This, combined with relatively fast pharmaceutical production, may be highly beneficial and convenient for the repeated treatments needed for chronic CVDs.

Several ncRNA-based therapies are under preclinical development, and a limited but potentially increasing number have reached clinical development for cardiovascular diseases [216]. Challenges related to RNA stability, delivery, and cardiac targeting remain. Current strategies mainly rely on two approaches. The first approach aims at inhibiting pathogenic ncRNAs or mRNAs, using complementary oligonucleotides including short interfering RNAs (siRNAs), antisense oligonucleotides (ASOs), antagomiRs, and miRNA sponges [217], which inhibit target interaction with other molecules or prompt their degradation. The second approach, instead, aims at restoring or enhancing endogenous protective functions of ncRNAs by delivering, for example, miRNA mimics, synthetic lncRNAs, or circRNAs [217].

Even if these approaches occasionally converge on similar regulatory outcomes, their mechanisms of action may differ. Therapeutic miRNAs and miRNA mimics (designed to reproduce their activity) recognize target transcripts mainly through an 8-nucleotide seed region, leading to translational repression or mRNA degradation depending on the degree of complementarity beyond the seed region [218]. Therapeutic interventions with other RNA-based strategies rely on different principles [218]. For instance, while siRNAs are typically designed to match their RNA target through the seed region and promote its cleavage through Argonaute, antagomiRs exploit complementarity to mature miRNAs to sequester or inhibit their function. ASOs, instead, bind their target through longer complementary regions and promote either RNAse-H based degradation or steric blockade. For the specific mechanisms involved in their pharmacological effect, we refer to more extensive reviews [218].

Among mechano-ncRNAs and mechanically associated ncRNAs involved in CVDs, microRNAs are the most extensively employed as therapeutic agents or targets, in contexts including atherosclerosis, myocardial infarction, heart failure, hypertension, and cardiac fibrosis (Table 5) [217]. An example of therapy based on mechano-miRNAs can be represented by the *miR-29* family, which is involved in the cellular responses to stiffness and in CVD pathologies such as hypertrophy and cardiac fibrosis [112,219,220]. In skin fibrosis, the *miR-29b* mimic Remlarsen reached clinical testing, where it has shown good safety and evidence of efficacy in Phase I/II clinical trials, reducing ECM deposition and scarring during wound healing [221]. Although the setting is clearly different, this offers a provisional rationale for investigating *miR-29* mimics also in other types of fibrosis, after dedicated validation. Therapeutic targeting of lncRNAs or circRNAs remains largely limited to preclinical studies, although the increasing understanding of their mechanisms of action is expected to accelerate the development of lncRNA-based interventions for future clinical application.

**Figure 5:**
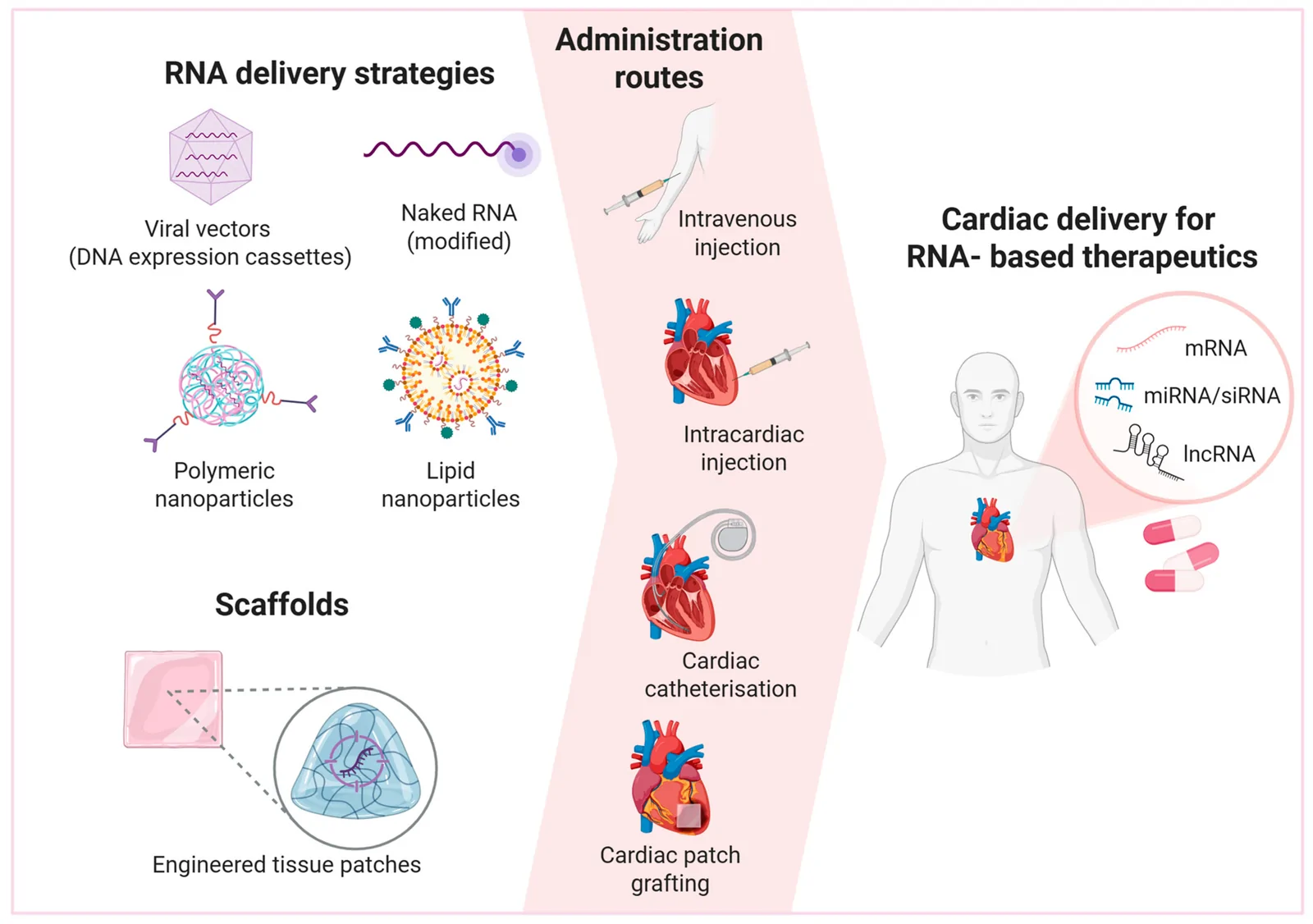
Delivery strategies to achieve cardiac targeting. RNA molecules can be delivered to the cardiovascular system with several types of formulations: as naked RNAs (modified for improved stability), or through viral vectors, dendrimers, polymeric nanoparticles, or lipidic nanoparticles. To improve stability and specificity of delivery, RNAs (naked or encapsulated) can be incorporated into tissue engineering scaffolds. Common administration routes for cardiac delivery of RNA therapeutics are intravenous injection, intracardiac injection, cardiac catheterization, or cardiac patch grafting.

A major obstacle to the clinical translation of RNA therapeutics is achieving efficient and selective delivery to the heart. Despite promising preclinical results, limited biodistribution, off-target accumulation, and poor cellular uptake often reduce efficacy in clinical settings [216,234]. Naked RNA molecules are negatively charged, hydrophilic, and unstable in aqueous environments, and generally larger than conventional small molecule drugs (Figure 5). Indeed, compared to the average weight of the latter (<1 kDa), RNA-based therapeutics are in the range of 4–14 kDa for ASOs and siRNAs, 200 kDa for single-guide RNAs (sgRNA) for CRISPR editing and 700–7000 kDa for mRNAs [216,235]. Consequently, their cell permeability is generally poor, but can be prompted with chemical modifications that drive pore formation, translocation, or endocytosis [236,237]. Downstream, RNA drugs risk rapid degradation by RNAses or by the lysosome [236,237]. Upstream, free RNA molecules may undergo fast systemic clearance through the liver and kidneys and degradation by other circulating RNAses [236,237]. Moreover, extracellular and intracellular RNAs may activate innate and adaptive immune responses, further limiting their therapeutic application [235].

To overcome these evolutionary measures against foreign and potentially harmful RNA molecules, and improve RNA drug stability and tissue-specific delivery, several strategies have been developed (Figure 5). The following sections aim to give a summary of the technical solutions to the cardiovascular delivery problem, taking into account that mechanically altered and stiff tissues may affect drug distribution, as in the case of cardiac fibrosis [238]. In general, the pharmaceutical delivery of RNA molecules for cardiovascular conditions is based either on direct chemical modification or on their encapsulation within carriers or scaffolds [218]. For the purpose of this review, the specific approaches to improve delivery through carriers will be explored, with all the mentioned ncRNA examples summarized in Table 5.

### 5.1. Viral-Based Cardiac Delivery

Among available delivery systems, viral vectors (e.g., adenovirus, lentivirus) remain among the most efficient platforms for nucleic acid transfer. With a capacity of up to 8 kb, nucleic acid cargos can be packaged into viral capsids and delivered with high transduction efficiency by exploiting the natural mechanisms of viral infection (Figure 5) [216].

Adeno-associated viruses (AAVs) are particularly attractive due to their tissue tropism, long-term expression, and relatively mild immune response compared with other viral systems [239]. AAVs do not deliver RNA molecules directly: the vector carries a DNA expression cassette that is transcribed in the nucleus of transduced cells, so that the therapeutic RNA (e.g., a miRNA, a short hairpin RNA, a miRNA sponge, or a lncRNA/circRNA) is produced intracellularly [240]. This allows sustained cargo expression, making AAVs promising for RNA-based therapeutics in chronic cardiovascular conditions, such as fibrosis and heart failure. Among adeno-associated vectors, AAV9 and AAV6 serotypes have shown marked cardiac tropism in animal models and are therefore applicable to heart-directed delivery in these systems [241]. For example, AAV9-mediated modulation of *miR-30d* has been successfully used in a mouse model of cardiac hypertrophy [222]. AAV5 has also shown positive outcomes in cardiac delivery, as it promoted efficient, homogeneous gene transfer in pig hearts, with low extra-cardiac distribution and no toxicity reported in that study [242].

However, the transition of AAV, and in general, viral vectors, into clinical practice carries important limitations due to the risks of immune activation and insertional mutagenesis, combined with high production costs. In addition, the optimal serotypes for selective cardiac targeting in humans remain to be fully defined in clinically relevant models, as tropism is often species- and serotype-dependent [243]. To propose solutions to the adverse effects of viral vectors, the engineering of more specific and serotype-optimized viral capsids and the development of non-viral alternatives are being actively pursued.

### 5.2. Nanoparticle-Based Cardiac Delivery

Nanoparticles (NPs) are among the most studied non-viral systems for RNA delivery, offering potentially improved safety profiles and greater control over biodistribution and release, compared with viral vectors [244]. These core-shell structures, typically smaller than 100 nm, can protect and transport therapeutic or diagnostic cargos, including RNA, and are produced through different physicochemical approaches (e.g., microfluidics, electrospinning) [244,245,246,247]. NP-based ncRNA delivery has been extensively explored in cardiovascular preclinical models [248], with major platforms including dendrimers, polymeric nanoparticles, and lipidic nanoparticles (Figure 5).

Dendrimers are highly branched synthetic structures that can be engineered for RNA encapsulation and targeted delivery [249,250]; they have been explored in different dense tissues, including the rodent heart [243]. For instance, dendrimers functionalized with oligo-arginines and cardiac Cell-Penetrating Peptides (CPPs) enabled preferential delivery to cardiomyocytes of siRNA anti-*AT1R* (encoding for Angiotensin II type 1 Receptor) in an Ischemia-Reperfusion (IR) rat model, improving cardiac recovery without evidence of toxicity [251]. However, concerns remain regarding long-term tolerability, particularly for polyamidoamine-based dendrimers [252,253]. Moreover, their clinical translation is limited by the frequent need for invasive administration, such as intracardiac injection [251], due to suboptimal systemic distribution.

Polymeric and lipidic nanoparticles (NPs) have been extensively investigated for cardiac delivery of RNA therapeutics [254,255]. Polymeric nanoparticles, of size 1–200 nm for optimized delivery [254], are typically formed by copolymers such as synthetic polymers (e.g., polyamidoamine, polyethylenimine or poly-L-lysine), natural polymers (e.g., chitosan), and a combination of the two (e.g., polylactic-co-glycolic acid complexed with chitosan) [243]. For instance, poly(D,L-lactic acid), poly(D,L-glycolic acid) (PLGA) provides biocompatible and biodegradable carriers with tunable release properties and targeting capabilities [243]. PLGA-based NPs have been successfully used for cardiac RNA delivery, including the systemic administration of *miR-30b-5p*-loaded particles functionalized with poly-ethylene glycol (PEG) and Cardiac Homing Peptide (CHP), which improved cardiac function and reduced infarct size in a rat MI model [255]. The tunable cargo release of PLGA-based NPs, which can undergo gradual hydrolysis into lactic and glycolic acids, and their favorable safety profile make them attractive for the treatment of chronic cardiovascular conditions; however, they offer limited tissue penetration and cellular uptake [256]. These challenges highlight the need for delivery systems with improved compatibility, particularly considering that mechanically altered cardiovascular environments may influence nanoparticle distribution and internalization.

Liposomes and lipid nanoparticles (LNPs) are RNA delivery systems composed of amphiphilic molecules that self-assemble into membrane-like structures, typically around 100 nm in size [257,258]. Their composition includes ionizable lipids that protect the RNA cargo, together with phospholipids, PEG-lipids, and cholesterol on the outer shell [259]. Their low cytotoxicity and scalable production make them attractive alternatives for cardiac RNA delivery, although biocompatibility depends on lipid composition, dose, and route of administration [240]. Among lipid-based carriers, extracellular vesicles (EVs) have gained attention due to their cell-derived origin, small size (diameter >30 nm), generally low immunogenicity, and reported ability to cross some biological barriers in preclinical models [248,260]. Although still at an early stage for clinical validation, the use of EVs in cardiovascular pathologies is a widely documented field of research at the preclinical level. For example, in rodent MI models, exosomes (small EVs of 30–150 nm in diameter) can deliver mimics of miRNAs that are mechanically associated or mechanoresponsive, including *miR-29b* [221,224], *miR-126* [225], *miR-146a* [225], and *miR-302* [226], improving cardiac repair after myocardial infarction by reducing fibrosis, inflammation, and cardiomyocyte damage. However, their limited cargo capacity remains a challenge, and biodistribution and targeting are difficult to control.

Liposomes and LNPs have been widely investigated for cardiovascular ncRNA delivery, with several applications summarized in other reviews [261] and mentioned in Table 5. In the context of mechanically-related CVDs, liposomes enabled systemic delivery of stress-responsive *miR-145* in a rabbit MI model, reducing infarct size and improving cardiac function [227]. Similarly, LNP-mediated delivery of the mechanically associated lncRNA *OIP5-AS1* (*CYRANO*), involved in pressure overload-induced heart failure, improved cardiac recovery in mouse models of Ischemia/Reperfusion injury [228]. Although most applications remain preclinical, the LNP-formulated siRNA drug Patisiran, approved by the FDA for hereditary transthyretin-mediated amyloidosis (hATTR), shows that LNP-siRNA formulations are feasible in humans for ncRNA delivery [229]. However, it acts by silencing hepatic transthyretin production, and therefore it lacks evidence of cardiac targeting. Moreover, although additionally proposed for treatment of hATTR-related cardiomyopathy, the therapeutic use of Patisiran in that context was not approved by the FDA for lack of significant cardiovascular improvement in the patients [230].

General disadvantages of lipidic vectors, shared with other carriers, include rapid plasmatic clearance, capture by the reticulo-endothelial system, and off-target accumulation in the filter organs [258]. As a result, systemic injections may target cardiac tissues poorly, creating the need for alternative, and unfortunately often more invasive, administration routes (e.g., intracardiac injections and catheterizations) (Figure 5). Alternative systems may involve the functionalization of the outer shell of the lipidic NPs to improve active targeting [262]. In addition, advanced tissue engineering scaffolds may be employed to act as reservoir platforms in cardiac grafts, facilitating targeted distribution of the therapeutic (Figure 5) [263].

### 5.3. Challenges and Perspectives of Clinical Translation

The clinical translation of mechano-ncRNA therapeutics for cardiovascular diseases still faces major challenges, particularly regarding delivery, specificity, and safety. For example, the cardiac environment, characterized by the low permeability of the sarcolemma and continuous pumping, limits the efficient delivery and retention of RNA therapeutics, especially after systemic administration [264]. Safety remains an important concern, as each delivery platform presents distinct limitations. Adeno-associated viral vectors may trigger innate and adaptive immune responses, whereas nanoparticles can induce unintended cytotoxicity depending on their chemical composition [217], requiring careful optimization for biocompatibility.

On another note, the presence of several splicing isoforms in target mRNAs and non-coding RNA transcripts increases the risk of off-target effects and reduced efficacy when using ncRNA-based strategies, highlighting the need for accurate isoform and subcellular characterization.

With this in mind, in clinical settings, some ncRNA-based therapies for CVDs have reached human trials, such as patisiran (although not approved for hATTR cardiomyopathy) [229,230] and *miR-132* inhibitor CDR132L [153]. CDR132L treatment in the CVD context was safe and elicited target engagement in a phase II trial after MI but did not meet its primary endpoint (change in left ventricular volume index), with exploratory signals in patients with advanced remodeling [153]. Other candidates, such as siRNA anti *NOX2* [231] or *CircSlc8a1* [232], have reached promising efficacy endpoints without raising significant adverse effects, but only in rodent models. Overall, evidence for clinical applications of targeted heart delivery remains limited [265].

Regarding specificity, in preclinical models, cardiotropic AAVs have shown more selective cardiac transduction than most systemically delivered nanoparticles, although tropism properties remain species-dependent and human data are limited. Cellular uptake of nanoparticles commonly occurs through endocytosis, but the exact mechanism is strongly dependent on the physicochemical properties [266]. Intracellular trafficking often results in endo-lysosomal degradation of both the carrier and its cargo unless endosomal escape mechanisms are incorporated (further details can be found in other reviews [236,237]).

Carrier delivery can be improved through passive or active targeting strategies. Passive targeting relies on physicochemical properties of the carrier and on features of the pathological tissue. Surface modifications that enhance circulation time, stability, and biodistribution, such as conjugation with cholesterol or vitamin E [261], or association with albumin or lipoproteins [243], fall into this category. Surface charge and hydrophilicity influence biodistribution; for example, the use of more hydrophilic moieties instead of cholesterol improves extrahepatic delivery, and preventing ApoE binding to LNPs promotes higher cardiac accumulation after intravenous administration in mice [267]. Nanoparticle composition additionally affects the formation of the protein corona, which can influence biodistribution, aggregation, and toxicity [268]. The pathological microenvironment also offers important opportunities for passive targeting. Following myocardial infarction, increased vascular permeability driven by VEGF can transiently enhance nanoparticle accumulation through the enhanced permeability and retention (EPR) effect (largely inferred from rodent models) [269,270]. However, the transient nature of the EPR effect makes it instrumental for early treatments, with a short action window [271]. Likewise, ischemic tissues display acidification (pH drops from 7.4 to 6.8) and increased protease activity (e.g., Matrix MetalloProteases—MMPs) [271,272], features that can be exploited using responsive biomaterials. In this context, tissue engineering scaffolds to encapsulate naked RNAs or vector-RNA complexes can enable a higher physico-chemical control on the release kinetics at the target tissue [273], while improving the stability of the formulations [274]. For example, protease-cleavable hydrogels have been used to deliver anti-*MMP2* siRNA to infarcted hearts, reducing adverse remodeling [275], while pH-sensitive hydrogels enabled controlled release of miRNAs and antagomiRs to cardiac tissue [276,277].

Active targeting exploits ligand-receptor or antibody-antigen interactions to direct carriers toward selected cell populations. In the heart, cardiomyocyte binding peptide (CMP), angiotensin 1 peptide (AT1), and ischemic myocardium targeting peptide (IMTP) have been used to improve cardiac delivery in vivo [278,279]. For example, IMTP-conjugated nanoparticles successfully delivered siRNAs against the lncRNA *AK156373* to infarcted myocardium [233], whereas CMP-functionalized lipid nanoparticles increased cardiomyocyte expression of *OIP5-AS1* [228]. Other approaches involve anti CD71-Fab fragments for cardiac delivery of siRNAs and ASOs [280], and hyaluronic acid functionalization to target CD44-expressing cells [281,282], which can be potentially applied in the cardiac context to specifically target CD44+ cells—mainly fibroblasts and immune cells in the heart [281].

Although systemic cardiac targeting is still suboptimal overall, recent studies show that cardiotropic lipid nanoparticles can accumulate in the heart in animal models and transiently modulate cardiomyocyte function after systemic administration [267]. Although validation in larger animals and humans remains necessary in many cases to expand the therapeutic claims, the cardiac RNA delivery field is advancing.

For mechanosensing-dependent CVDs, combining RNA carriers with mechanically or pathologically responsive targeting strategies may improve therapeutic precision and limit off-target effects; mechanoresponsive nanomedicines, mostly sensitive to shear stress, have been reviewed in [283]. In mechanically altered conditions in the heart, the dysregulated profibrotic activation of cardiac fibroblasts determines the onset of fibrosis, a common hallmark of several CVDs that represents a path to heart failure and also a barrier to the therapies meant to treat it. Fibrotic tissue is stiff and dense, rich in cross-linked collagens, and its altered architecture hinders the penetration, distribution, and cellular uptake of delivery vectors [284]. The same limitation has been described in other fibrotic settings: for example, in pancreatic cancer, a densely fibrotic microenvironment reduces delivery efficiency, and the EPR effect is insufficient, a limitation termed the “fibrotic barrier” to nanomedicine [285]. Cardiac-specific aspects of nanotechnology for fibrosis are covered in [286]. The above-mentioned concept creates a paradox for mechano-ncRNA therapeutics: the mechanical and structural alterations of the tissue would limit biodistribution of the therapeutic agents exactly in the area that should be targeted.

Two complementary strategies are the most proposed in cancer and general fibrosis [285]. The first modulates the fibrotic process through stromal cell ablation, reprogramming, or alteration of the metabolism; it involves the co-delivery, with the nanomedicine of choice, of inhibitors of profibrotic biochemical signals (e.g., TGFβ) or ECM remodeling (e.g., MMP inhibitors), to reduce fibroblast activation and indirectly ameliorate tissue permeability. Although theoretically elegant, this approach is limited because delivery of the antifibrotic agent can itself be hampered by the fibrotic barrier, and because targeting directly pathways such as TGFβ can elicit different effects in non-stromal cells and induce resistance [285]. The second approach optimizes the carrier systems to achieve responsiveness to mechanically altered environments, or to actively target functional receptors enriched in fibrotic tissues. Mechanical susceptibility can be achieved, for instance, by tuning carrier composition, shape, size, and stiffness. Mechano-responsive moieties, such as the large conductance ion channels (MscL), or specific ligands for ECM molecules can be functionalized on the surface to promote accumulation in the stressed, fibrotic areas [283,287,288]. Alternatively, tissue engineering scaffolds encapsulating the nanomedicine, such as hydrogels, allow more invasive but specific local delivery, bypassing systemic transport [273,274,275,289].

These approaches could enable spatiotemporal controlled delivery of mechano-ncRNAs or their inhibitors to sites exposed to altered stiffness, compression, strain, osmotic stress, or flow. However, further optimization of carrier sensitivity and specificity will be required before clinical translation, particularly in chronic diseases characterized by persistent low-intensity mechanical stress.

## 6. Conclusions and Future Perspectives

In recent years, ncRNAs have emerged as central regulators of gene expression, cellular identity, and intercellular communication, far beyond their original view as transcriptional by-products. Their ability to control chromatin organization, transcription, RNA processing, translation, and intercellular signaling places them at the core of both physiological development and disease pathogenesis. This work reviews the growing evidence that defines the critical role of mechano-responsive ncRNAs (here called mechano-ncRNAs) in cardiovascular disease (CVD), mediating vascular, valvular, and myocardial remodeling, as well as in cellular responses to mechanical cues. In this context, mechano-ncRNAs may function as molecular memory elements, converting acute or chronic biomechanical stimuli (e.g., matrix stiffness, strain, altered flow) into long-lasting transcriptional and epigenetic programs that ultimately contribute to CVD progression. Given their functional versatility, mechano-ncRNAs may represent an emerging niche at the intersection of mechanobiology and cardiovascular medicine that creates a significant opportunity for novel translational outcomes.

The integration of in vitro and in vivo mechanobiology models with multi-omic and computational approaches is accelerating the discovery of mechano-responsive ncRNA signatures with potential diagnostic and therapeutic value. However, functional validation remains essential to define their biological relevance and clinical applicability. The confirmation of causal, human-relevant targets remains a challenge; many ncRNAs are poorly conserved across species, which often limits extrapolation from animal models. Another challenge is represented by the development of delivery systems capable of achieving efficient, selective, and durable delivery of ncRNA-based treatments to cardiovascular tissues.

Therapies based specifically on mechano-ncRNAs still remain an underexplored field in cardiovascular research, where they could provide a tailored strategy to address more specifically those pathological processes in CVDs that are induced by dysregulated mechanosensing. Future advancements may allow the development of delivery platforms specifically responsive to the cardiovascular mechanical environment, with improved cell type specificity, and careful consideration of the context-dependent functions of ncRNAs across different cardiac cell populations. While most evidence remains preclinical and important challenges remain (particularly regarding specificity, delivery and safety), continued progress in ncRNA biology and cardiac delivery systems may open new avenues for precision medicine in cardiovascular disease.

## Figures and Tables

**Table 1:** Representative mechanically associated ncRNAs related to CVDs.

	Name	Type	Disease with Mechanical Component	Target	In Vitro	In Vivo	Ref.
Involved in mechanotransduction	*miR-133*	miRNA	Cardiac hypertrophy (chronic mechanical overload)	Downregulation of *CTGF*, decreasing collagen production	Mouse CFs	Rat cardiac hypertrophy model	[107]
	*miR-30*	miRNA	Cardiac hypertrophy (chronic mechanical overload)	Downregulation of *CTGF*, decreasing collagen production	Mouse CFs	Rat cardiac hypertrophy model	[107]
	*miR-34*	miRNA	Myocardial infarction (increased fibrosis and wall stress); pressure overload (TAC)	Downregulation of vinculin, Notch1, Sema4b, Pofut1 and Bcl6 in the cardiac context.	Human cancer cells and mouse CMs	Mouse models of MI and TAC	[108]
	*miR-29*	miRNA	Myocardial infarction (increased fibrosis and wall stress)	Inhibition of mRNAs involved in ECM remodeling	Mouse CFs	Mouse model of MI	[109]
	*miR-33*	miRNA	Heart failure (increased fibrosis and wall stress); pressure overload (TAC)	In CFs it regulates cardiac remodeling by preserving lipid raft cholesterol and inducing MMP16/MAPK	Mouse CFs	Rodent models of MI and TAC	[110,111]
	*circHelz*	circRNA	Myocardial infarction (increased fibrosis and wall stress)	In CFs, NFATC2-driven *circHelz* facilitates YAP nuclear translocation, promoting fibrosis. In CMs, it suppresses *miR-133-3p*, activating pyroptosis.	Human CFs and CMs	Mouse model of MI	[112,113]
	*circNfix*	circRNA	Myocardial infarction (increased fibrosis and wall stress)	*circNfix* controls the YAP interactor Ybx1.	Mouse and rat CMs	Mouse model of MI	[114]

Abbreviations: CF, cardiac fibroblasts; CM, cardiac myocytes; MI, myocardial infarction; TAC, transverse aortic constriction; ECM, extracellular matrix.

**Table 2:** Representative mechanoresponsive ncRNAs implicated in response to stiffness in CVDs.

	Name	Type	Stimulus	Direction of Change	Target/Effect	In Vitro	In Vivo	Ref.
Related to ECM stiffness	*miR-21*	miRNA, mechanoeffector	Matrix stiffness Cyclic stretch; Laminar shear stress	Increased	Induced by MRTF-A; inhibits anti-fibrotic genes	Human MSCs	Rat wound healing model	[123]
	*CYTOR*	lncRNA, mechanoresponsive	Matrix stiffness	Decreased	Regulates gene expression in response to stiffness; Modulates *miR-155/NF-kB*	Human MSCs; mouse CMs	Mouse cardiac hypertrophy model	[124,125]
	*NEAT1*	lncRNA, mechanoresponsive	Matrix stiffness	Increased	Promotes apoptosis in CMs; drives fibrotic transformation repressing SMAD7 in CFs	Human and rodent vSMCs, CMs and CFs	Rodent models of MI, TAC, and coronary occlusion	[126,127]
	*MALAT1*	lncRNA, mechanoresponsive (possibly mechanoeffector)	Vascular matrix stiffness	Increased	Modulates vSMCs proliferation and migration, and neointima formation	Human and rodent vSMCs, CMs and CFs	Rodent models of MI, TAC, and coronary occlusion	[128]

Abbreviations: MSCs, mesenchymal stem cells; CM, cardiac myocytes; CF, cardiac fibroblasts; vSMCs, vascular smooth muscle cells; MI, myocardial infarction; TAC, transverse aortic constriction.

**Table 3:** Representative mechanoresponsive ncRNAs implicated in response to strain and pressure overload in CVDs.

	Name	Type	Stimulus	Direction of Change	Target/Effect	In Vitro	In Vivo	Ref.
Related to increased strain	*let-7 family* *(let-7a)*	miRNA, mechanoresponsive; let-7a: candidate mechanoeffector	Cyclic stretch	Dysregulated	Let-7a: anti-hypertrophic, directly reduces CaM expression	Neonatal rat ventricular myocytes	Rodent model of AngII-induced cardiac hypertrophy	[146,147]
	*miR-208a*	miRNA, mechanotransducer	Cyclic stretch	Increased	Via TGF-β1 signaling, promotes endoglin and collagen I expression	Rat H9c2 cardiac myoblasts	Rat model of volume overload	[148,149]

Abbreviations: CaM, calmodulin; AngII, angiotensin II.

**Table 4:** Representative ncRNAs implicated in mechanical response to altered flow conditions in CVDs.

Name	Type	Stimulus	Direction of Change	Target/Effect	In Vitro	In Vivo	Ref.
*miR-93*, *miR-484*	miRNA, mechanotransducer	OSS (vs. pulsatile flow)	Increased in OSS	Targets KLF2 and eNOS	HUVECs	Elevated in sera of patients with coronary artery disease	[161]
*miR-712*	miRNA, mechanoeffector	OSS	Increased	Inhibits TIMP3, activating MMPs, prompting atherosclerosis	Mouse arterial ECs	Mouse model of atherosclerosis	[162]
*miR-21*	miRNA, mechanoeffector	LSS	Increased	Modulates apoptosis and eNOS activity	HUVECs		[131]
*LASSIE*	lncRNA, mechanosensor	LSS	Increased	Induced by KLF2/KLF4, it associates with junctional and cytoskeletal proteins	Human ECs	Mouse model of adventitial damage	[104]
*LEENE*	lncRNA, mechanoeffector (provisional)	LSS	Increased	Induced by KLF2/KLF4, recruits RNA Pol II to eNOS promoter	Human ECs	Mouse model of adventitial damage	[163]
*AF131217.1*	lncRNA, mechanotransducer	LSS	Increased	It sponges *miR-128-3p*, indirectly regulating KLF4, eNOS, VCAM and ICAM	Human ECs		[164]
*circRNA-LONP2*	circRNA, mechanoeffector (provisional)	LSS	Decreased	It sponges *miR-200a-3p*, promoting Keap1/YAP1/EZH2, blocking NFE2L2/HMOX1; oxidative stress and inflammation	Human ECs	Mouse model of atherosclerosis	[165]
*CircRNA-CZFP292*	circRNA, mechanotransducer	LSS	Levels not directly affected	It binds SDOS, allowing flow-induced alignment through cytoskeleton and focal adhesions	Human ECs	Mouse cZfp292 knock-out	[166]

Abbreviations: HUVEC, human umbilical vein endothelial cells; ECs, endothelial cells.

**Table 5:** Discussed ncRNAs targeting strategies for pre-clinical or clinical development.

ncRNA	Type	Administration Route	Vector	Subtype	In Vivo CVD Setting	Clinical Translation	Ref.
*miR-30d*	miRNA mimic	Systemic intravenous (IV) injection	Viral vector	Adeno-associated Virus serotype 9	Mouse model of cardiac hypertrophy	-	[222]
*miR-30b-5p*	miRNA mimic	Intracardiac injection	Polymeric nanoparticle	PLGA-PEG-CHP	Rat model of MI	-	[223]
*miR-29b*	miRNA mimic	Microneedle patch in the infarct zone	Exosome		Mouse model of MI	-	[221,224]
*miR-126*	miRNA mimic	Intramyocardial injection	Exosome	Alginate hydrogel encapsulating exosome-miR-126	Rat model of MI	-	[225]
*miR-146a*	miRNA mimic	Systemic injection (IV)	Exosome	Milk-derived exosomes conjugated with IMTP	Rat model of ischemia/riperfusion (I/R)	-	[225]
*miR-302*	miRNA mimic	Systemic injection (IV)	Exosome	Exosome from bone marrow-derived stem cells (BMSC), functionalized with CMP	Rat model of ischemia/riperfusion (I/R)	-	[226]
*miR-145*	miRNA mimic	Systemic injection (IV)	Lipidic nanoparticle	Liposomes	Rabbit model of MI	-	[227]
*OIP5-AS1 (CYRANO)*	MiRNA mimic	Systemic injection (IV)	Lipidic nanoparticle	LNP-Oip5-as1-CMP	Mouse model of ischemia/reperfusion (MI/R)	-	[228]
*Patisiran (siRNA anti-hATTR)*	siRNA	Systemic injection (IV)	Lipidic nanoparticle	LNP-PEG-C-DMG-DLin-MC3-DMA-siRNA hTTA	-	Proposed for treatment of restrictive cardiomyopathy of hereditary transthyretin-mediated amyloidosis (hATTR) (Not approved)	[229,230]
*CDR132L*	synthetic antisense oligonucleotide miR-132 inhibitor	Systemic injection (IV)	N.A.	Stabilized by chemical modification, locked nucleic acid (LNA)	-	Human trials phase IIb for heart failure (primary endpoint not met)	[151,152,153]
*NOX2-siRNA*	siRNA	Intramyocardial injection	Acid-sensitive nanoparticle	Polyketal NPs	Mouse model of MI	-	[231]
*CircSlc8a1*	siRNA		Viral vector	Adeno-associated Virus serotype 9	Rodent model of cardiac hypertrophy from pressure-overload	-	[232]
*AK156373*	siRNA		Nanoparticle		Mouse model of MI	-	[233]

## Data Availability

No new data were created or analyzed in this study. Data sharing is not applicable to this article.
